# An Atypical Heterojunction in Favor of Conversion of CO_2_ and Sunlight into C_2_H_4_

**DOI:** 10.1002/advs.202503336

**Published:** 2025-05-08

**Authors:** Qin He, Dongge Ma, Yangyang Du, Qiang Huang, Jianfei Ji, Xu Wang, Hongwei Ji, Wanhong Ma, Jincai Zhao

**Affiliations:** ^1^ Key Laboratory of Photochemistry Institute of Chemistry Chinese Academy of Sciences Beijing National Laboratory for Molecular Sciences Beijing 100190 P. R. China; ^2^ University of Chinese Academy of Sciences Beijing 100049 P. R. China; ^3^ Department of Chemistry College of Chemistry and Materials Engineering Beijing Technology and Business University Beijing 100048 P. R. China

**Keywords:** C‐C Coupling, charge Transfer, CO_2_ Photoreduction, photocatalysis, reaction mechanisms

## Abstract

Current heterojunction semiconduction assemblies, including type I, II, Z‐Scheme, and S‐Scheme constructures, enable the utilization of longer‐wavelength sunlight for photocatalytic conversions. However, such benefits are often achieved at the expense of either the redox potentials of the conduction and valence bands or the quantum yield due to additional electron–hole recombination across the heterojunction interface. Herein, an atypical type II heterojunction constituted of Au/TiO_2_/MFU‐4l is reported that demonstrates outstanding catalytic performance in photocatalytic reduction of carbon dioxide (CO_2_) to ethylene (C_2_H_4_) through tuning up‐converting of holes in MFU‐4l component raised from full‐spectrum solar irradiation. Anchored to the edge of cube MFU‐4l with a TiO_2_ cover layer, aurum ions (Au^+^)supported by aurum (Au) nanoparticles enables such a reverse hole‐transfer event through leveraging the Ti‐O^−•^‐Au^+/0^‐^•−^O‐Zn potential, which significantly accelerates the hole‐dominated oxidative desaturation of C‐C intermediates from CO_2_ reduction into C═C bond products. The catalyst efficiently converts CO_2_ to C_2_H_4_ with more than 90% selectivity and a yield of 107.0 µmol g^−1^ h^−1^ under simulated sunlight. Electron paramagnetic resonance (EPR) experiments directly observe the holes formed in visible‐light excited MFU‐4l moiety of Au/TiO_2_/MFU‐4l that are fused into TiO_2_ component's holes, thereby generating more hydroxyl radicals (•OH) than that TiO_2_ is excited alone under ultraviolet (UV) carbon dioxide (CO_2_) light of the same intensity.

## Graphical abstract

An atypical heterojunction is constructed in the Au/TiO_2_/MFU‐4l catalyst to achieve visible light‐induced hole reverse transfer in the valence band of MFU‐4l, thereby facilitating the desaturation of hole‐dominated CO_2_ reduction reaction intermediates to the C═C bond products. This design enhances the efficiency of photocatalytic CO_2_ conversion to C_2_H_4_ products.

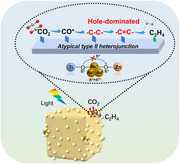

## 1 Introduction

The photocatalytic reduction of carbon dioxide (CO_2_) using solar energy for large‐scale production of high value‐added chemicals such as carbon monoxide (CO), methane, methanol, ethane, ethylene (C_2_H_4_), and so on is considered a common goal for the next generation of mankind.^[^ ^1^, ^2^, ^3^, ^4^ ^]^ Among these C products, C_2_H_4_ and other unsaturated hydrocarbons are, of course, the most desired ones as the raw material for polyolefin, but the evolution of C_2_^+^ products is one of the most challenging aspects since it needs subtle channel designed not only to couple into the saturated C─C bond followed by the commonly difficult start‐up step of CO_2_ activation but also to operate oxidative desaturation of C─C single bonds. A lot of heterogeneous composite photocatalysts have been developed to reduce CO_2_ to C_2_^+^ products upon a broader solar spectrum to visible or even near IR light, but the utilization efficiency of visible light and the selectivity of olefins production have not been satisfactory since the insight into the structure‐effective relationship has not been completely clear.^[^ ^5^, ^6^, ^7^ ^]^ For example, which heterogeneous structures, type II or Z‐Scheme, have more benefits for producing unsaturated C_2_^+^ products other than C_1_? Moreover, is there any synergistic effect between visible or near‐infrared (near‐IR) light and ultraviolet (UV) light excitation in the solar spectrum that enhances photocatalytic performance in these heterojunction structures, and if so, why? Especially, what real role of the frequently‐used noble modification on the energy band of heterojunction structures to enhance the efficiency and selectivity of C_2_^+^ plays has not been accurately correlated to date.^[^ ^8^, ^9^ ^]^ Clearly, understanding these issues will be very beneficial for designing and developing more effective catalysts to produce bulk chemicals C_2_H_4_ directly by converting CO_2_ and focused sunlight in the future.

**Scheme 1:**
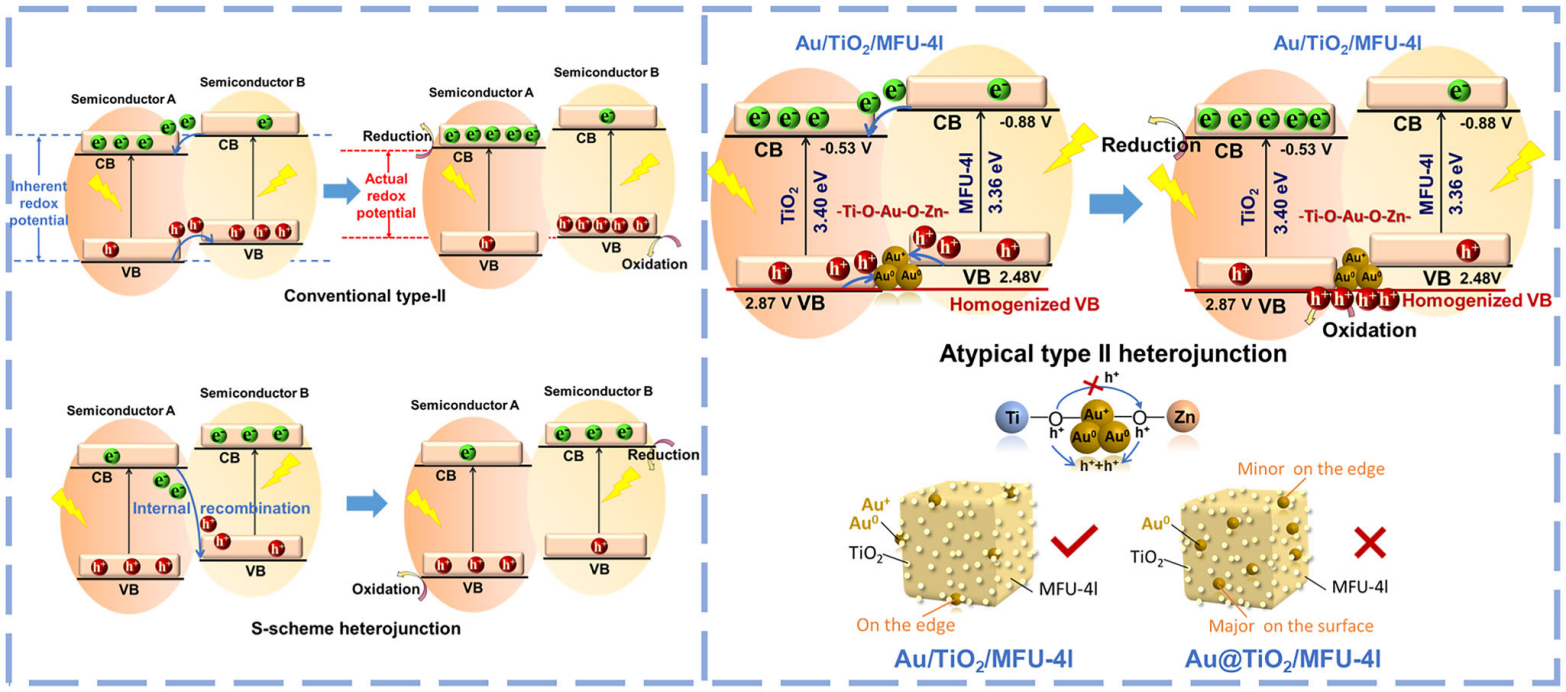
Schematic of electron and hole transfer mechanisms in atypical type II heterojunctions, compared with conventional type II and S‐heterojunctions.

Currently, most photocatalysts that can produce C_2_^+^ products are multi‐component heterojunction materials,^[^ ^10^, ^11^ ^]^ and the use of precious metal‐loaded modification is also very common,^[^ ^8^, ^12^ ^]^ with the aim of facilitating electron transfer and C‐C coupling in CO_2_ reduction reaction (CO_2_RR). The design of heterojunction materials is mainly to broaden the absorption of the solar spectrum and enhance the utilization efficiency of solar energy, as shown in **Scheme** **1**.^[^ ^13^, ^14^ ^]^ However, a fatal drawback of this approach is that the inherent redox potential or quantum efficiency of the photocatalysts must be sacrificed once the heterojunction is formed; namely, i) the redox ability of the valence band and conduction band is weakened in conventional type II heterojunction (Scheme 1, top left), and ii) the excited electrons in one component are artificially forced to recombine with the holes formed in another component, resulting in a mandatory decrease in the quantum yield (Scheme 1, bottom left), i.e., so‐called S‐heterojunction model. Despite maintaining their respective optimal oxidation and reduction capacity, such an S‐heterojunction avenue for electron‐hole recombination will significantly waste a large portion of the excited carriers to accomplish this operation.

The deposition of a few percent noble metal (platinum, aurum (Au), Argentum, etc.) nanoparticles on the heterogeneous catalyst has long been a proven approach to reduce the activation barrier of CO_2_ by resulting in high‐energy “hot electrons” from local surface plasmon resonance effect (LSPR),^[^ ^15^, ^16^, ^17^ ^]^ which can use visible‐light irradiation without reducing the redox potential of photocatalysts too. It shows significant effects whether it is located on inorganic semiconductor materials or organic ones such as g‐C_3_N_4_,^[^ ^18^, ^19^ ^]^ metal organic framework (MOF),^[^ ^20^ ^]^ and even covalent organic framework.^[^ ^21^, ^22^ ^]^ Especially, it was recently found that the heterojunction materials inorganic/MOF combinations after loading precious metal particles as photocatalysts were more conducive to the selective generation of C_2_^+^ from CO_2_RR. Therefore, TiO_2_ as a “star” inorganic semiconductor has been proven to be able to be coupled with some MOF materials to improve the CO_2_RR activity under UV light irradiation.^[^ ^23^, ^24^, ^25^, ^26^ ^]^ In these examples, although TiO_2_’s conduction band is mainly used as an excellent electron staging base for promoting CO_2_ reduction, its ability to simultaneously oxidize the C─C single bond for desaturation by the parent hole of TiO_2_ will suffer heavy losses due to the transfer into incompetent MOF's valence band along conventional type‐II heterojunction mechanism.^[^ ^27^, ^28^ ^]^ In this way, we envision that the introduction of noble metals to a proper position between TiO_2_/MOF heterojunction can make broader solar spectra to improve photocatalytic efficiency without sacrificing either TiO_2_’s inherent hole performance or excitation efficiency. That is, is there a suitable MOF material as a candidate moiety of heterojunction as well as a unique loading of precious metal to evolve into a new‐style heterojunction with a reversed flow of holes (see Scheme 1 right)?

Herein, we finally found such a MOF, MFU‐4l (Zn_5_Cl_4_(BTDD)_3_, where BTDD is bis‐(1H‐1,2,3‐triazolo‐[4,5‐b],[4′,5′‐i])dibenzo[1,4]dioxin), the four units of tetra‐coordination Zn^2+^‐N_3_Cl surround one units of six‐coordination Zn^2+^‐N_6_ without oxygen coordination. When we used an in situ growth of MFU‐4l with H_2_BTDD as a linker and ZnCl_2_ solution in the presence of TiO_2_ nanoparticles to prepare TiO_2_/MFU‐4l heterostructure, the resulting TiO_2_/MFU‐4l exhibits still a classical type II heterostructure property. It, astoundingly, turns into the atypical type II heterojunction that we look forward to while we use the photo‐deposition method to reduce AuCl_4_^−^ into some Au^+^ supported with most Au nanoparticles (Au NPs) that must be loaded on the edge region of the TiO_2_/MFU‐4l quasi‐cube since remained Au^3+^ ions are preferentially anchored by the Ti─O bond and Zn─Cl bond suspended at the edge of TiO_2_/MFU‐4l. Along the possible hole transfer channel, Ti‐O^−•^‐Au^+/0^‐^•−^O‐Zn, this Au/TiO_2_/MFU‐4l construction is once irradiated by the simulated sunlight (wavelength 350–1000 nm), the photo‐induced holes on the MFU‐4l component under visible‐light part irradiation should be extracted by the Au NPs containing Au^+^ ion at specific sites and reversely fused into the more positive holes raised from the valence band of TiO_2_ component under the UV part excitation. The move was a switch to leverage on the fewer potential holes of MFU‐4l component to make oxidation potent available immediately to key desaturation of C‐C intermediates of CO_2_RR into C═C product, significantly improving the CO_2_ olefination activity and selectivity. In situ electron paramagnetic resonance (EPR) spectra directly observed the enhancement of signals of 5,5‐dimethyl‐1‐pyrroline N‐oxide‐hydroxyl radicals (DMPO‐•OH) generated from water oxidation by TiO_2_‐hole upon simulated sunlight irradiation. We argued that, by the transformation of, more holes formed in MFU‐4l moiety were elevated through O^−•^ lattice vibration exchange between Ti‐O^−•^‐Au^+/0^‐^•−^O‐Zn enhanced by Au^+^/Au nanoparticles anchored on the edge (see Scheme 1 right). This composite photocatalyst avoids the defects of common type II or S‐heterojunction systems and provides much more favorable features for hole‐mediating desaturation of C‐C intermediates into C═C products.

## 2 Results and Discussion

### 2.1 Synthesis and Characterization of Au/TiO_2_/MFU‐4l

**Figure 1:**
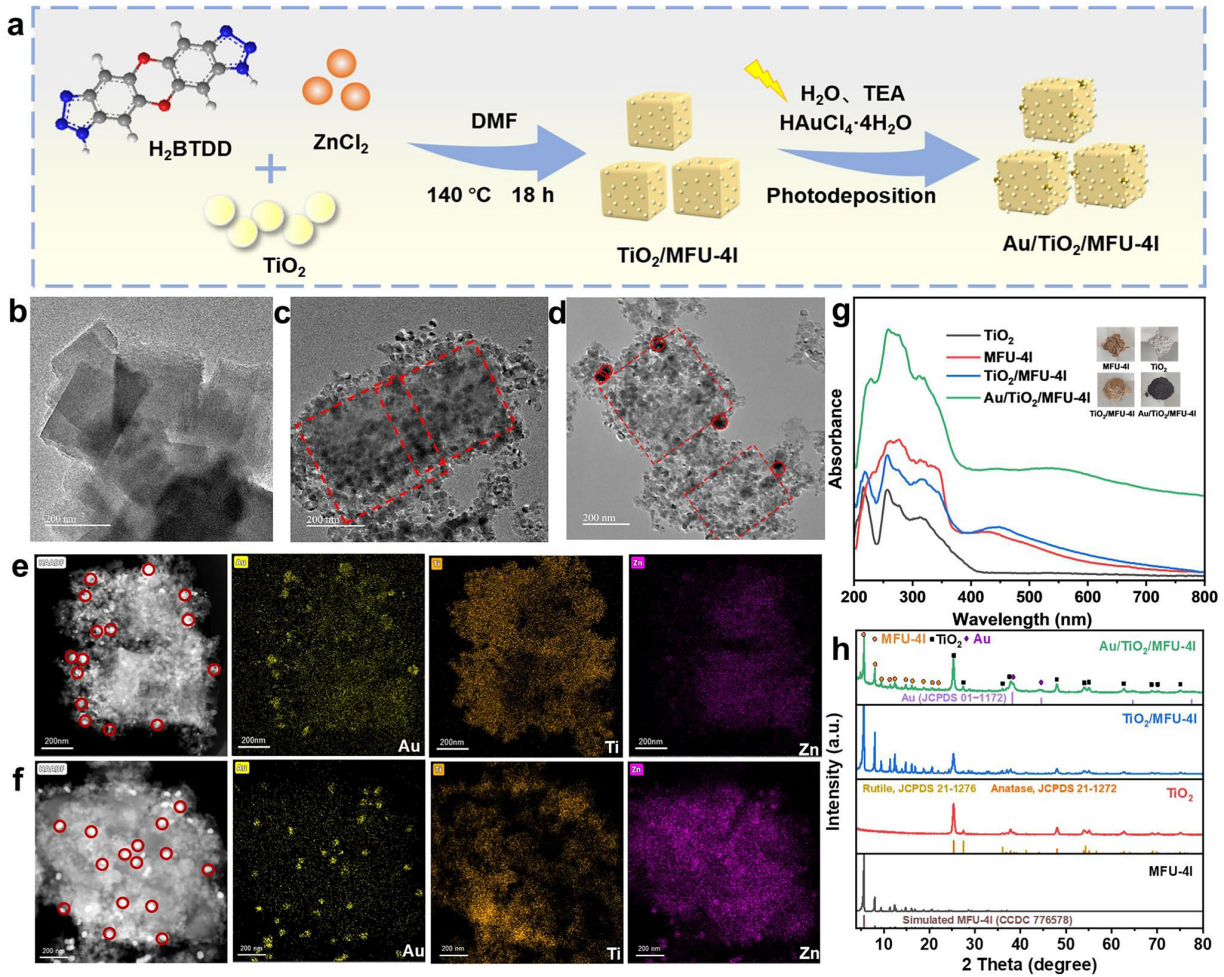
a) Illustration of the synthetic process of Au/TiO_2_/MFU‐4l composite material. The TEM images of b) MFU‐4l, c) TiO_2_/MFU‐4l, d) Au/TiO_2_/MFU‐4l. High‐angle annular dark‐field (HAADF) and EDX elemental mapping images of e) photochemically‐deposited Au/TiO_2_/MFU‐4l target sample and f) physical deposition of Au NPs on TiO_2_/MFU‐4l to prepare Au@TiO_2_/MFU‐4l controlled sample. g) UV–vis DRS spectra and h) XRD patterns of different samples.

Au/TiO_2_/MFU‐4l is obtained through in situ hydrothermal synthesis of MFU‐4l with H_2_BTDD as a linker and ZnCl_2_ in the presence of P25‐TiO_2_ nanoparticles (**Figure** **1**a). The mass ratio of TiO_2_ to MFU‐4l of the feed was controlled within the range of 0.42‐2.5. Subsequently, Au nanoparticles were post‐deposited on the surface of TiO_2_/MFU‐4l by photochemical‐reduction of HAuCl_4_ solution to obtain Au remained some Au^+^ on the edge region of TiO_2_/MFU‐4l composite materials. Meanwhile, for comparison, the pure MFU‐4l sample was prepared with identical conditions but in the absence of the TiO_2_ component. The structural morphologies of the materials were characterized by transmission electron microscope (TEM) (Figure 1b–d) and scanning electron microscope (SEM) (Figure S1, Supporting Information). Pure MFU‐4l has a cubic block‐like structure with a size of ≈200 nm. The smaller nanoparticles of P25‐TiO_2_ with an average diameter of ∼25 nm irregularly cover the surface of MFU‐4l, and there are no TiO_2_ particles wrapped inside of MFU‐4l. This MFU‐4l component feature in the TiO_2_/MFU‐4l was very consistent with the previous preparation of MFU‐4l without TiO_2_, indicating MFU‐4l's excellent ability to grow independently. Post‐deposition of Au nanoparticles on the surface of TiO_2_/MFU‐4l exhibited the larger Au nanoparticles with an average diameter of≈60 nm. The High‐angle annular dark‐field (HAADF) images and corresponding element mapping images (Figure 1e; Figure S2, Supporting Information) of Au/TiO_2_/MFU‐4l confirm the unique structure of the sample, where Au nanoparticles are almost exclusively anchored at the edge of TiO_2_/MFU‐4l cubic block‐like materials. This is significantly different from the case of physically depositing Au nanoparticles that have been prepared in advance to obtain the Au@TiO_2_/MFU‐4l where Au nanoparticles were well‐proportioned dispersed on each face of the cuboidal TiO_2_/MFU‐4l construction (Figure 1f). Moreover, the edge deposition of Au NPs on TiO_2_/MFU‐4l differs from the cases where Au is anchored on pure MFU‐4l or TiO_2_ alone, as achieved by the photochemical reduction of HAuCl_4_ solution. This special distribution of Au nanoparticles can be summarized as the result of the synergistic effect of two key factors: i) the anchoring of TiO_2_ onto MFU‐4l leaves a small number of strongly coordinated Cl^−^ sites unsubstituted at the edge region, while the central region is mainly occupied by TiO_2_; and ii) the Au precursor, anionic AuCl_4_^−^, undergoes diffusion and subsequent photo‐reduction by both photo‐active TiO_2_ and MFU‐4l components under UV light irradiation. As illustrated in Figure S3 (Supporting Information), during this process, the exposed Cl^−^ sites in the edge region of quasi‐cubic TiO_2_/MFU‐4l can be forcibly replaced by AuCl_4_^−^ units more preferentially than those in the central region. The results of subsequent experiments will prove that this Au‐loading at the edge of heterojunction may cause the new pathways of hole transfer between TiO_2_ and MFU‐4l to benefit C═C formation from an oxidative desaturation of C─C single bond intermediates during photocatalytic CO_2_RR (see later). The addition of Au was ≈8% wt of the total Au/TiO_2_/MFU‐4l composite, but the outcome significantly changed the basic property of MFU‐4l with a high specific surface area (SSA). It brings the ≈3429.6 m^2^ g^−1^ SSA of pure MFU‐4l down to only 480.8 m^2^ g^−1^ of Au/TiO_2_/MFU‐4l (Table S1, Supporting Information) through determination by N_2_ adsorption‐desorption isotherms (Figure S4, Supporting Information), while assembling of 125% wt TiO_2_ to MFU‐4l surface only results in 1610.7 m^2^ g^−1^ SSA. Considering that the pore structure of MFU‐4l remains intact (Figure 1h), the notable decrease in SSA after Au loading is likely due to a combination of factors. The formation of large Au nanoparticles at the edge regions may alter the pore structure and entrance morphology at the TiO_2_/MFU‐4l interface. Additionally, residual triethylamine and its oxidation products generated during the photodeposition process may partially occupy the pores of the MFU‐4l framework, thereby contributing to the observed decrease in SSA. More interesting, Au loading raises this assembly's exact opposite adsorption behavior between CO and C_2_H_4_ relative to its parent MFU‐4l and TiO_2_/MFU‐4l (see later Figure 5), which we believed to be the main origin of high C_2_H_4_ selectivity during its mediating photocatalytic CO_2_RR. Moreover, thermogravimetric analysis (TGA) was used to confirm that the thermal stability of Au/TiO_2_/MFU‐4l is ≈450 °C (Figure S5, Supporting Information), which is coincident with previous reports and indicates MFU‐4l as an excellent MOF material with high thermal stability.^[^ ^29^, ^30^ ^]^

The overall light absorption of as‐prepared Au/TiO_2_/MFU‐4l is significantly stronger than other single components and TiO_2_/MFU‐4l, and it covers a wider range of the solar spectrum, especially upon loading Au nanoparticles (Figure 1g). About 8% Au loading imparts a distinct black coloration to the entire catalyst (Figure 1g, inserts), providing a very favorable basis for harnessing more solar energy.

**Figure 2:**
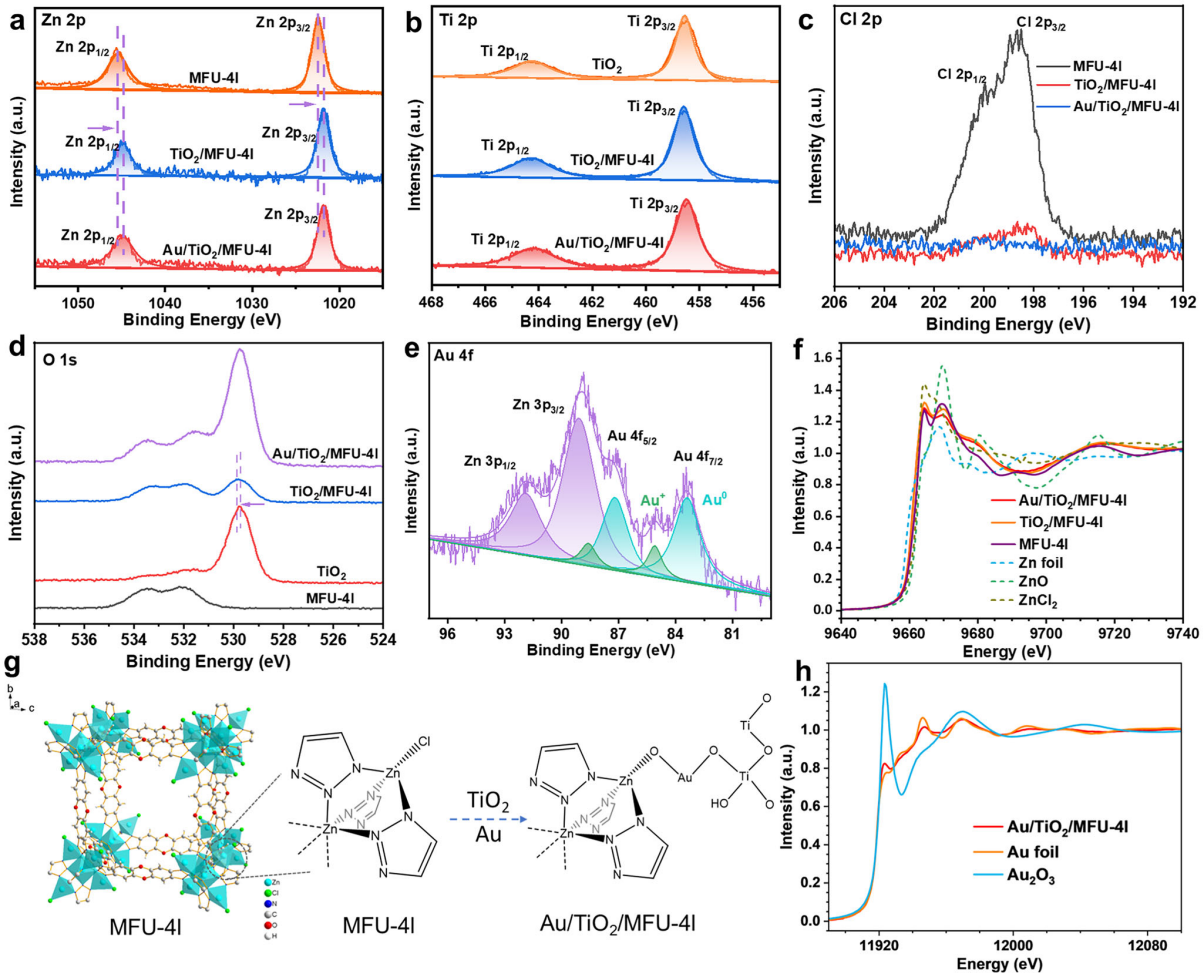
XPS spectra of a) Zn 2p, b) Ti 2p, c) Cl 2p, d) O 1s, e) Au 4f. (f) Zn K edge XANES spectra g) Crystal structure of MFU‐4l and schematic diagram after forming Au/TiO_2_/MFU‐4l composite material. h) Au L3 edge XANES spectra.

Further, the crystal structure of the Au/TiO_2_/MFU‐4l composite material and its monomer samples were analyzed by X‐ray diffraction (XRD) (Figure 1h). The most important thing here is to focus on the MFU‐4l and Au NPs aspects since both are formed in situ. The composite material clearly displays the characteristic diffraction peaks of both MFU‐4l and TiO_2_ as main components, indicating that the presence of TiO_2_ NPs did not hinder the formation of the MFU‐4l main ordered structure. In particular, the XRD pattern of the MFU‐4l component in our Au/TiO_2_/MFU‐4l is consistent with both our control pure MFU‐4l and previous reports,^[^ ^31^ ^]^ indicating its facile synthesis and good crystallinity of MFU‐4l moiety even pre‐existing suspended TiO_2_ NPs. The diffraction peaks of Au/TiO_2_/MFU‐4l composite material at 38.3° and 44.6° distinctly corresponded to the (111) and (200) characteristic crystal planes of Au nanoparticles, respectively,^[^ ^32^ ^]^ indicating the successful reduction of Au from the Au^3+^ solution. More detailed information on the chemical valence states of the as‐prepared Au/TiO_2_/MFU‐4l composite and its monomer was analyzed by X‐ray photoelectron spectroscopy (XPS) (**Figure** **2**a–e). As opposed to a more normal Ti, Zn 2p spectra, there are two characteristic XPS events, namely, O 1s and Cl 2p XPS manifestation, to strongly indicate the bonding sites between TiO_2_ and MFU‐4l component: i) pure MFU‐4l with the normal broad peak at 198 eV corresponding to the binding energy of 2p electrons in Cl, indicating that a part of Zn^2+^ in the tetrahedral structure coordinates with three BTDD^2−^ ligands and one Cl^−^ ion (Figure 2g).^[^ ^31^, ^33^ ^]^ However, after the formation of TiO_2_/MFU‐4l heterojunction, the signal of the Cl 2p peak was significantly reduced and disappeared completely once upon loading Au NPs (Figure 2c). This clearly demonstrated the substitution of Cl in MFU‐4l by some more nucleophilic groups that only be attributed to TiO_2_ possessed, most possibility, dangling oxygen on the surface of TiO_2_ component; ii) in pure MFU‐4l, there is nothing about metal‐coordinated O 1s peak at 529.7 eV but only the covalent oxygen of xanthene ring of BTDD linker at 532  and 533.5 eV. After the formation of TiO_2_/MFU‐4l heterojunction, a peak at 529.9 eV, similar to the octahedral coordination Ti‐O of the TiO_2_ component did appear, just responding to the removal of the Cl element linked to tetra‐coordinated Zn site. We argued this oxygen site in replace of Cl to bond with Zn, while leaving its other end to bond to other possible sites, for example, dangling Ti‐O^−^ site of TiO_2_ moiety surface or Au^+^‐O^−^ site. As for the valence state of loaded Au on the TiO_2_/MFU‐4l heterojunction, its 4f XPS feature shows 83.4 , 87.2 eV, and 85.1 , 88.6 eV, which are attributed to the 4f binding energy of Au^0^ and Au^+^ species, respectively (Figure 2e),^[^ ^34^ ^]^ which is in agreement with the subsequent Au L3 edge X‐ray absorption near edge spectroscopy (XANES) results.

To further reveal which possible bonding to the Zn‐O‐ end after Cl removal, namely, Zn‐O‐Ti sites or Zn‐O‐Au‐O‐Ti, we performed X‐ray absorption near edge spectroscopy (XANES) and extended X‐ray absorption fine structure (EXAFS) analysis (Figure 2f; Figure S6, Supporting Information). According to our fitting results (Figures S7 and S8 and Table S2, Supporting Information), the Zn‐Cl of Zn^2+^ in TiO_2_/MFU‐4l disappears, and the Zn‐N/O coordination number increases once TiO_2_ is loaded, which is attributed to the dangling oxygen on the surface of TiO_2_ replacing the Cl in the tetradentated ZnN_3_Cl and generating to the ZnN_3_O structure. Furthermore, the result of Au L3 edge XANES and EXAFS (Figure 2h; Figures S9 and S10 and Table S2, Supporting Information) confirmed the formation of Au nanoparticles and the simultaneous existence of typical Au^+^‐O^−^ coordination in the Au/TiO_2_/MFU‐4l composite. In comparison with the physical deposition of Au NPs distributed on the terraces without Au^+^ formation (Figure S11, Supporting Information), these results suggest that such Zn‐O‐Au‐O‐Ti sites can only exist at the edge region of every Au/TiO_2_/MFU‐4l quasi‐cube since special need of Au^+^ supported by Au NPs to bond between TiO_2_ and MFU‐4l synchronously, which may create a unique interface for the photocatalytic CO_2_RR into C═C products.

### 2.2 Photocatalytic CO_2_RR Performance

**Figure 3:**
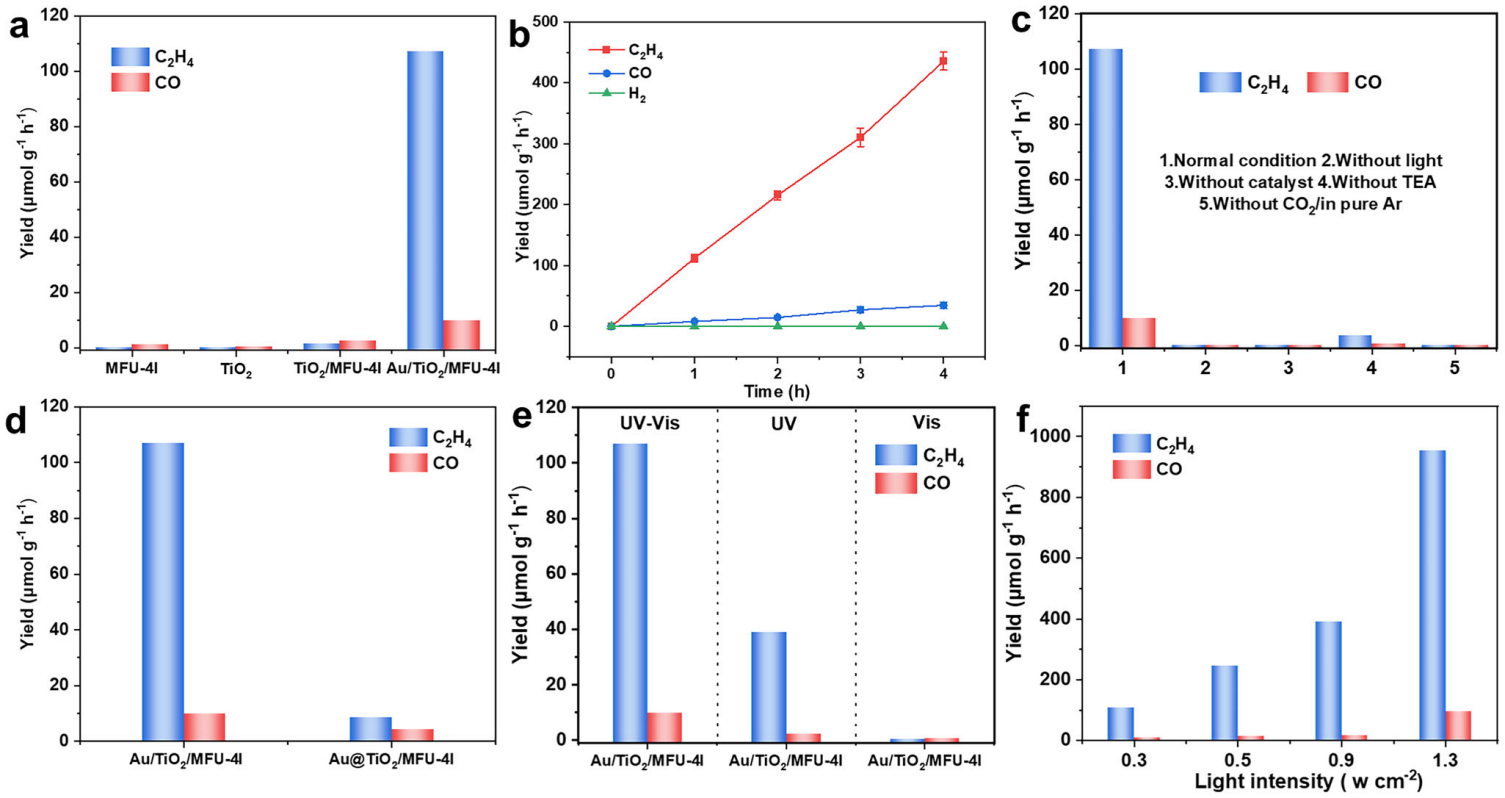
a) Yield of products in photocatalytic CO_2_ reduction reaction of different samples under full‐spectrum irradiation with an intensity of 0.3 W cm^−2^. b)Time‐dependent C_2_H_4_, CO, and H_2_ evolution of Au/TiO_2_/MFU‐4l. c) The effect of reaction conditions on photocatalytic reduction of CO_2_. d) Performance testing of Au@TiO_2_/MFU‐4l prepared by direct deposition of Au NPs. e) Performance testing under different wavelength light sources (UV–vis–NIR (0.3 W cm^−2^): 350–1000 nm, UV (0.03 W cm^−2^): 365 nm, vis–NIR (0.27 W cm^−2^): 420–1000 nm) and f) different light intensities in full spectrum illumination.

The photocatalytic activity of as‐prepared Au/TiO_2_/MFU‐4l for CO_2_RR was evaluated under full‐spectrum irradiation of simulated sunlight (**Figure** **3**). In a typical process, 5 mg of the catalyst and 100 µL of triethylamine (TEA) as a hole‐sacrificial reagent were added to 20 mL of water solution in an 80 mL photo‐reactor. Then, the reaction mixture was degassed with pure CO_2_ to remove air for at least 30 min, and the photocatalytic system was filled with one atmosphere of CO_2_ and sealed. A 300 W xenon lamp was used as the light source of simulated sunlight. Under irradiation for 3 h, only a small amount of CO was detected when we used either of the two monomers, TiO_2_ and MFU‐4l, as photocatalysts to perform CO_2_RR (Figure 3a). After the formation of heterojunction materials, the CO_2_ photocatalytic activity of TiO_2_/MFU‐4l was slightly enhanced, and a small amount of C─C coupling products began to appear beside CO. Gratifyingly, the CO_2_ photoreduction activity and C═C selectivity were significantly enhanced upon deposition of Au nanoparticles on the edge regions of TiO_2_/MFU‐4l quasi‐cube particles, and no liquid products such as methanol or ethanol were detected, while gaseous products primarily consisted of substantial amounts of ethylene and trace amounts of CO (see Figure S12, Supporting Information). After optimizing the relative content (wt%) of TiO_2_ and Au NPs to MFU‐4l main structure (Figure S13, Supporting Information), 8%Au/125%TiO_2_/MFU‐4l exhibited both excellent C_2_H_4_ yield of 107.0 µmol g^−1^ h^−1^ and selectivity as high as 91.5% (Figure 3b), and stood out in most of reported photocatalytic CO_2_ reduction examples for C_2_H_4_ production(Table S4, Supporting Information).^[^ ^35^, ^36^, ^37^, ^38^, ^39^, ^40^, ^41^, ^42^ ^]^ Notably, no by‐product hydrogen (H_2_) was generated during the CO_2_RR with our Au/TiO_2_/MFU‐4l photocatalyst. In contrast, H_2_ was the main product when using the control Au/TiO_2_ photocatalyst without MFU‐4l (Figure S14, Supporting Information). Obviously, the introduction of the MFU‐4l component in the Au/TiO_2_/MFU‐4l effectively avoids conventional H^+^ coupled electron transfer on zero‐valent Au^0^ NPs that always causes the awkward side‐reaction of H_2_ evolution. To determine the source of C_2_H_4_, a series of control experiments were conducted, and almost no products were detected in the reaction system without light, catalyst, or CO_2_ cases (Figure 3c). In addition to using triethanolamine as a sacrificial agent, we further performed photocatalytic CO_2_RR using other sacrificial agents (e.g., triethanolamine, ascorbic acid), and the results all detected the production of C_2_H_4_ (Figure S15, Supporting Information). Moreover, isotope labeling experiments using ^13^CO_2_ under identical conditions confirmed that the result in the main product ^13^C_2_H_4_ and minor ^13^CO exclusively came from ^13^CO_2_ (Figure S16, Supporting Information). In addition, the Au/TiO_2_/MFU‐4l catalyst showed good stability in several cycles without an obvious decrease in activity (Figure S17, Supporting Information).

A series of control experiments were carried out to verify the functions of each component in Au/TiO_2_/MFU‐4l. When variable metal ions (Cu^2+^, Ni^2+^) partially replace the tetrahedron central metal ion Zn^2+^ in MFU‐4l (Figure S18, Supporting Information), the yield of C_2_H_4_ is lower than that of intrinsic MFU‐4l afforded in Au/TiO_2_/MFU‐4l. However, when non‐variable valence metal ion Cd^2+^ was used to partially replace Zn^2+^, the photocatalytic activity was only slightly reduced, indicating the crucial role of non‐variable valence Zn^2+^‐based MFU‐4l in C‐C coupling and its desaturation into the double bond. To investigate the role of Au in this process of CO_2_RR into C_2_H_4_ main product, we conducted another control experiment using Au@TiO_2_/MFU‐4l (its morphology features see Figure 1f) as a reference prepared by directly depositing an equal amount of well‐reduction Au NPs in advance, then observed its C_2_H_4_ production under other photocatalytic conditions identical to Au/TiO_2_/MFU‐4l case. Sure enough, there was little photocatalytic CO_2_RR occurrence (Figure 3d). This shows that only Au nanoparticles containing some Au^+^ must be loaded on the edge region of TiO_2_/MFU‐4l, and it can play a role in producing C_2_H_4_ from photocatalytic CO_2_RR. Such a peculiar Au^+^/Au NP location‐dependent activity in Au/TiO_2_/MFU‐4l obviously does not follow the traditional type II mechanism in which the role of Au NP mainly mediates the electron transfer between two conduction bands and accelerates proton‐coupled electron transfer (PCET) process for protons reduction into hydrocarbons. Moreover, a series of reactions irradiated with different wavelengths but strictly controlled the same incident light intensity (0.3 W cm^−2^) to investigate our Au/TiO_2_/MFU‐4l performance of C_2_H_4_ production (Figure 3e). There was nearly little CO_2_RR to occur when using a light source with a wavelength more than 420 nm by which TiO_2_ moiety was rarely excited, indicating that to obtain the C_2_H_4_ product, the excitation of TiO_2_ moiety in Au/TiO_2_/MFU‐4l is necessary. However, the CO_2_ photocatalytic activity of the Au/TiO_2_/MFU‐4l catalyst under 365 nm UV light significantly decreased compared to full‐spectrum irradiation, even with exactly the same intensity (0.03 W cm^−2^) as the UV light part in the full‐spectrum illumination case. This violates the conventional dependence of wavelength‐quantum efficiency, by which normal UV‐photocatalysts such as TiO_2_, SrTiO_3,_ et al. always lose their quantum efficiencies with shifting the incident light wavelength toward visible light region.^[^ ^43^ ^]^ Since both LSPR excitation of Au nanoparticles and MFU‐4l excitation in the visible light excitation alone barely work (see Figure 3e right), such a remarkably concerted effect raised from UV and visible light should not belong to the classic type‐II heterojunction mechanism. In this regard, we tentatively define Au/TiO_2_/MFU‐4l as an atypical type II heterojunction, which will be discussed in detail later. Further, we investigated the effect of incident light intensity on the reaction through simulated solar spectrum irradiation (Figure 3f). It exhibited that the yield of C_2_H_4_ mediated by Au/TiO_2_/MFU‐4l photocatalyst was exponentially improved with the increase of light intensity. It is suggested that this performance could use as much as possible focused sunlight regardless of the usual photocatalytic self‐quenching drawbacks.

### 2.3 Characteristics of the Atypical type II Heterojunction and its Dominating Oxidative Desaturation of C‐C Intermediates

We highly inclined that the C═C bond formation depends on a very strong oxidation capacity of the photo‐induced hole rather than the reduction potential of conduction band electron on Au/TiO_2_/MFU‐4l since no matter from which channel of reaction, C═C double bond evolution from CO_2_ is more complicated than C─C singlet bond coupling. This requires more powerful holes on Au/TiO_2_/MFU‐4l, particularly in the use of the visible part of the sunlight. Thus, we used EPR to observe the DMPO trapping reaction of the photo‐induced hole in this Au/TiO_2_/MFU‐4l heterojunction.^[^ ^44^, ^45^ ^]^

**Figure 4:**
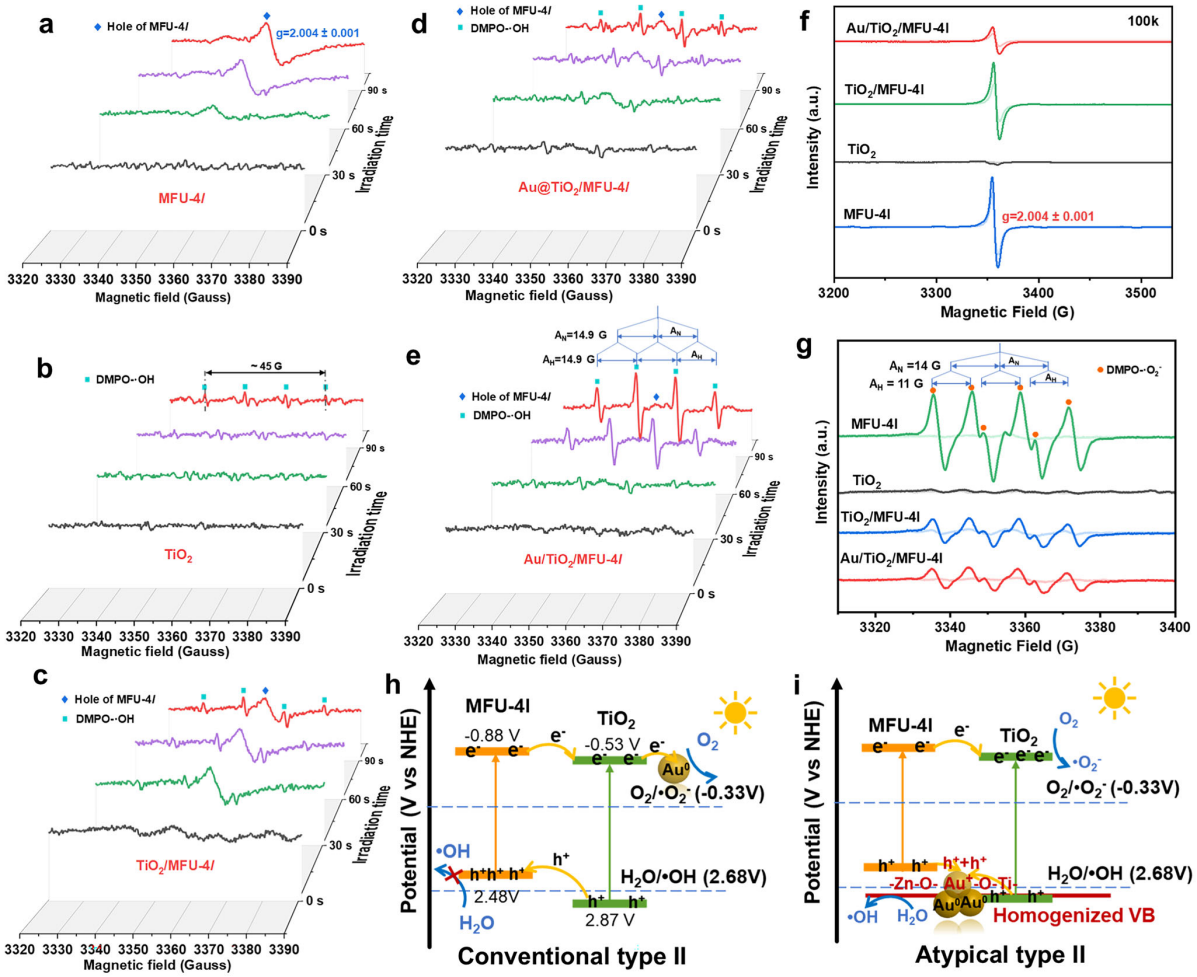
EPR spectra of DMPO ‐•OH in aqueous solution under different illumination times: a) MFU‐4l, b) TiO_2_, c) TiO_2_/MFU‐4l, d) Au@TiO_2_/MFU‐4l, e) Au/TiO_2_/MFU‐4l. f) EPR spectra of different materials on illumination under low‐temperature (100k), where light‐colored lines indicate measurement under dark conditions and dark‐colored lines indicate measurement under conditions after 2 min of illumination. g) EPR spectra of DMPO ‐^•^O_2_^−^ in methanol solution, where light‐colored lines indicate measurement under dark conditions and dark‐colored lines indicate measurement under conditions after 2 min of illumination. Charge transfer mechanism of h) conventional type II and i) atypical type II.

From the DMPO‐•OH EPR spectra observed at room temperature (**Figure** **4**a–e) upon simulated sunlight irradiation, the pure MFU‐4l component only showed the signal of its own organic radicals, no DMPO‐•OH signal arises due to the oxidation potential of photo‐generated holes on its valence band is lower than H_2_O/•OH (+2.68 V vs NHE).^[^ ^46^ ^]^ After forming a composite material with TiO_2_, the DMPO‐•OH signal began to emerge but was very weak, comparable to the TiO_2_ component alone, indicating that TiO_2_/MFU‐4l did not form a traditional type‐II heterojunction at this time. Namely, there is neither the increase of the hole signal of the MFU‐4l component nor the decrease of the DMPO‐•OH signal generated by the TiO_2_ component excited under UV part irradiation, confirming no hole transfer from TiO_2_ to MFU‐4l along a traditional type‐II heterojunction mechanism. Very interesting, when we used the Au@TiO_2_/MFU‐4l sample prepared by direct deposition of Au NPs on TiO_2_/MFU‐4l to perform the measurements of the DMPO‐•OH signals in this photocatalytic system (Figure 4d), a certain amount of DMPO‐•OH signal appeared and was gaining strength with the irradiation time relative to TiO_2_ alone (Figure 4b), while the g≈2.004±0.001 signal of MFU‐4l component's hole becomes gradually weaker than that of MFU‐4l alone (Figure 4a). Despite that the majority of Au NPs were not anchored in the particular edge of TiO_2_/MFU‐4l, a curious sign of the somewhat reverse hole transport from MFU‐4l to TiO_2_ moiety mediated by a small of Au located on the edge of TiO_2_/MFU‐4l has emerged, which did not follow the traditional type‐II (Figure 4h). More definitely, as Au/TiO_2_/MFU‐4l heterojunction prepared through the photochemical‐reduction of AuCl_4_^−^ on the special edge region of this quasi‐cube was used to observe the hole reaction in the presence of DMPO solution under simulated sunlight irradiation, it showed the strongest DMPO‐•OH radical signal among all used composite Au@TiO_2_/MFU‐4l, TiO_2_/MFU‐4l or monomer, while the g≈2.004 signal of MFU‐4l component's hole completely disappeared with extending irradiation and, never to be seen again (Figure 4e). At this time, it appeared reasonable to put this disappearance of photo‐induced holes in the MFU‐4l moiety down to merging into DMPO‐•OH radical in the TiO_2_ moiety through possible lattice vibration exchange of Ti‐O^−•^‐Au^+/0^‐^•−^O‐Zn. As‐resulted hole oxidation potential is enhanced even under visible‐light part irradiation and is the same as that of TiO_2_ moiety excited by the UV part of simulated sunlight. These results suggest that the photo‐generated charge transfer mechanism in the Au/TiO_2_/MFU‐4l heterojunction is not the conventional type II or S‐Scheme heterojunction, and herein we tentatively defined it as an atypical type II heterojunction (Figure 4i). Furthermore, the EPR signals of the samples without the addition of DMPO solution upon illumination under low‐temperature conditions (100k) were tested (Figure 4f). The EPR signal of the organic free radicals of MFU‐4l could be clearly observed, and there was no remarkable change in TiO_2_/MFU‐4l, whereas the sign of hole (g = 2.027) in TiO_2_ alone was very weak due to the very small UV excitation. However, the sign of the MFU‐4l’ hole was significantly weakened in Au^+^/Au on the edge region of TiO_2_/MFU‐4l cube‐like, while the sign of the TiO_2_ component’ hole was increased accordingly. This is consistent with the results of the free radical capture experiment at room temperature, confirming the formation of this atypical heterojunction. That is, coupling with visible‐light irradiation, photo‐generated holes possess the stronger potential to perform oxidative dehydrogenation or elimination reaction from favorable ‐HC‐CH‐, OHC‐CH‐ etc. intermediates of CO_2_RR. In addition, the electron behaviors on the conduction bands for both monomers and composites produced under the simulated sunlight were observed by the 5,5‐dimethyl‐1‐pyrroline N‐oxide‐superoxide radicals (DMPO‐^•^O_2_^−^) EPR experiments (Figure 4g). The intensity of the DMPO‐^•^O_2_^−^ signal created in the Au/TiO_2_/MFU‐4l case was much lower than that of the MFU‐4l alone case, suggesting the reduction of O_2_ remarkably decreased after electron injection from MFU‐4l into TiO_2_. Instead, the Au/TiO_2_/MFU‐4l generates the highest C_2_H_4_ yield among all other TiO_2_/MFU‐4l composites or monomers MFU‐4l, TiO_2_, indicating that these reduction steps of CO_2_RR by conduction band electrons were not the rate‐limiting step of C_2_H_4_ formation. To further verify the efficiency of charge separation and transfer, photoluminescence (PL) and time‐resolved photoluminescence (TRPL) spectroscopy were performed. As shown in Figure S21 (Supporting Information), Au/TiO_2_/MFU‐4l exhibits the lowest photoluminescence intensity compared to the other two materials, indicating the highest carrier separation efficiency. This observation deviates from the typical behavior of S‐scheme heterojunctions, which generally exhibit low carrier separation efficiency due to the forced recombination of electrons (e^−^) on the A component and holes (h^+^) on the B component of the heterojunction (see Scheme 1). Combined with the direct ESR evidence indicating the hole potential beyond that of a conventional type‐II heterojunction, our Au/TiO_2_/MFU‐4l system can be classified as an atypical type‐II heterojunction. Furthermore, the time‐resolved photoluminescence spectra of Au/TiO_2_/MFU‐4l show an average lifetime of 0.17 ns, which is shorter than that of TiO_2_ (0.34 ns) and TiO_2_/MFU‐4l (0.34 ns), suggesting that the incorporation of Au reduces the migration barrier for photo‐induced holes (see Scheme 1 right).^[^ ^47^ ^]^

**Figure 5:**
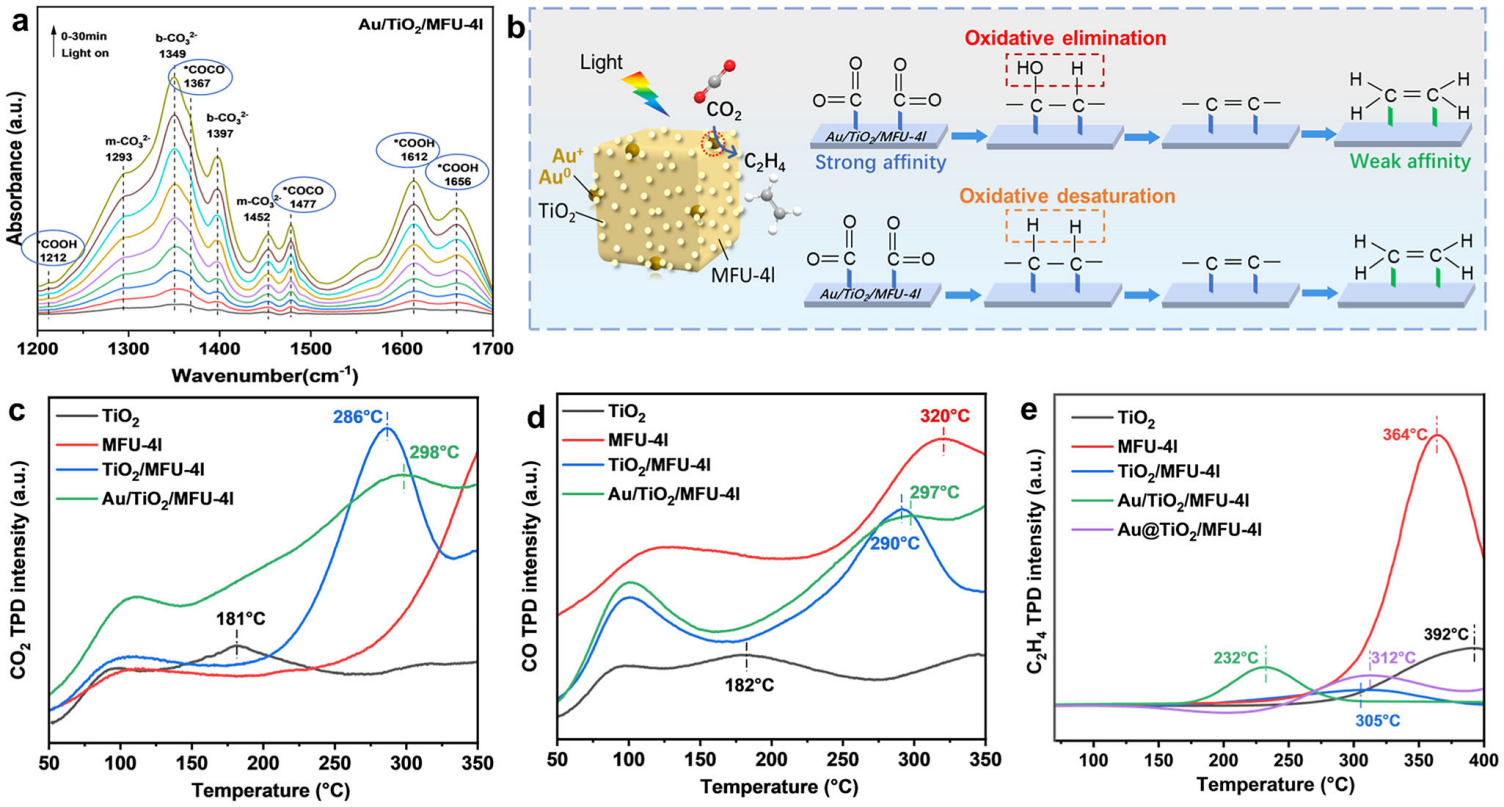
a) In situ FTIR spectra on the Au/TiO_2_/MFU‐4l photocatalyst for CO_2_ photoreduction process under irradiation. b) Illustration of photocatalytic reactions on the surface of Au/TiO_2_/MFU‐4l. c) CO_2_‐TPD, d) CO‐TPD, e) C_2_H_4_‐TPD profiles of different samples.

To unclose why the enhanced hole's oxidation ability of Au/TiO_2_/MFU‐4l favors C_2_H_4_ evolution in the photocatalytic CO_2_RR, in situ attenuated total reflection‐fourier tranform infrared (ATR‐FTIR) measurements were conducted to investigate the metastable intermediates of C‐C coupling in the photocatalytic CO_2_RR. Under the simulated sunlight irradiation, the in situ ATR‐FTIR spectra of Au/TiO_2_/MFU‐4l indeed showed the characteristic peaks at 1367 and 1477 cm^−1^ that were distinctly attributed to ^*^O═C─C═O, which very readily formed and was very stable like the most commonly initial C_1_ intermediate ^*^COOH species (**Figure** **5**a).^[^ ^48^, ^49^ ^]^ More important, there are no stable ^*^CO intermediate species formed on the Au/TiO_2_/MFU‐4l surface, suggesting our Au/TiO_2_/MFU‐4l with too remarkable reactivity toward formed ^*^CO to escape as an end‐product. Therefore, we contrasted the adsorption properties of Au/TiO_2_/MFU‐4l toward CO and end product C_2_H_4_ through temperature‐programmed desorption (TPD) measurements. As shown in Figure 5c–e, significantly different from any other individual component or TiO_2_/MFU‐4l composite or physically mixed Au@TiO_2_/MFU‐4l materials, once Au^+^/Au NP was loaded on the edge region, Au/TiO_2_/MFU‐4l exhibited just opposite bonding ability between CO and C_2_H_4_ molecules. The latter showed the weakest interaction with the surface, while the former did the strongest. Obviously this is very beneficial for deeply converting CO intermediate and quickly removing the C_2_H_4_ end‐product from the catalyst surface. In addition, we also measured the H_2_‐TPD profiles for these different materials (Figure S24, Supporting Information). The binding ability of the Au/TiO_2_/MFU‐4l samples to H_2_ is also stronger than that of pure TiO_2_ and Au/TiO_2_, which is consistent with the experimental observation of no competitive H_2_ evolution in this photocatalytic CO_2_RR. The high selectivity toward C_2_H_4_ rather than CH_3_CH_2_OH and C_2_H_6_ is attributed to the formation of an atypical heterojunction, which provides more powerful photogenerated holes than common heterojunctions. This facilitates both the C─C bond coupling and subsequent oxidative desaturation processes. Even if CH_3_CH_2_OH or C_2_H_6_ is formed as an intermediate, it will eventually be highly oxidized and dehydrated or dehydrogenation to C_2_H_4_ (^*^CH_3_CH_2_OH → C_2_H_4_ + H_2_O or ^*^CH_3_CH_3_ → C_2_H_4_ + H_2_). These results show that on the Au/TiO_2_/MFU‐4l interface, the ^*^O═C─C═O coupling intermediates readily form and stabilize, which facilitates the reductive hydrogenation into ─COH─CH─ or ─CH─CH─ and further oxidative elimination or desaturation into C═C product. In such a complex process from CO_2_ to C_2_H_2_, the desaturation of the C─C single bond into the C═C double bond is the most possible rate‐limiting step. Therefore, promoting both the oxidative ability and quantity of photo‐induced holes is obviously conducive to the smooth implementation of the process. On this point, the vast majority of previous designs and constructions of photocatalysts aimed at CO_2_RR into olefins have ignored this desaturation function by the photo‐induced holes and simply considered them as oxidizing sacrificial agents to provide protons. According to the above analysis, the possible CO_2_RR into the C_2_H_4_ pathway on Au/TiO_2_/MFU‐4l photocatalyst is proposed in Figure 5b.

## 3 Conclusion

An Au/TiO_2_/MFU‐4l composite photocatalyst is successfully prepared by in situ growth of MFU‐4l in the presence of TiO_2_ NPs and post‐deposition of Au nanoparticles on the edge region of as‐prepared quasi‐cube, which presents effective and selective conversion of CO_2_ into C_2_H_4_ under simulated sunlight irradiation. As‐prepared Au/TiO_2_/MFU‐4l photocatalyst displays as much as 107.0 µmol g^−1^ h^−1^ yield of C_2_H_4_ under 300 mW cm^−2^ stimulated sunlight and increases to≈1000 µmol g^−1^ h^−1^ yield of C_2_H_4_ as the intensity of the incident stimulated sunlight is four‐fold increase. TEM, SEM, and EPR trapping experiments observed that some Au^+^ supported by Au nanoparticles are mostly located in the edge region of TiO_2_/MFU‐4l quasi‐cube bulk which establishes an atypical type II heterojunctions to enable the excitation of MFU‐4l moiety from visible‐light parts with the same hole oxidation potential as TiO_2_ moiety excited by UV parts. With the established extraordinary desorption of the aimed C_2_H_4_ product and strong affinity to C_1_ intermediate on this unique valence band construction, our results support that conversion of CO_2_ into C═C products is easier to achieve by hole‐oxidation‐dominating the desaturation of ─C─C─ intermediates than that of conduction band electron reduction from the traditional type‐II heterojunction.

## Conflict of Interest

The authors declare no conflict of interest.

## Supporting information



Supporting Information

## Data Availability

The data that support the findings of this study are available in the supplementary material of this article.
