# Supplementary material for: An Atypical Heterojunction in Favor of Conversion of CO2 and Sunlight into C2H4
Source: Adv Sci (Weinh). 2025 May 8;12(27):2503336. doi: 10.1002/advs.202503336 (PMC12279171; doi:10.1002/advs.202503336)
Supplement: Supplementary file 1 — Supporting Information [file ADVS-12-2503336-s001.docx]

Supplementary Information

**An Atypical Heterojunction in Favor of Conversion of CO_2_ and Sunlight into C_2_H_4_**

**Authors:** Qin He, Dongge Ma*, Yangyang Du, Qiang Huang, Jianfei Ji, Xu Wang, Hongwei Ji*, Wanhong Ma*, and Jincai Zhao

***Corresponding author Email:**

whma@iccas.ac.cn, hwji@iccas.ac.cn, madongge@btbu.edu.cn.

**Experimental Procedures**

**Materials**

1,7-dihydrodibenzo[b,e][1,4]dioxino[2,3-d:7,8-d']bis([1,2,3]triazole) was purchased from Jilin Chinese Academy of Sciences - Yanshen Technology. Zinc chloride (ZnCl_2_), CuCl_2_⋅2H_2_O, NiCl_2_⋅6H_2_O, and triethylamine (TEA) were purchased from Sigma–Aldrich. Titanium oxide (TiO_2_) was purchased from Acros Organics. Sodium citrate and triethanolamine (TEOA) were purchased from Aladdin. Hydrogen tetrachloroaurate was purchased from Alfa Aesar. N, N-Dimethylformamide (DMF), methanol (MeOH), and dichloromethene (CH_2_Cl_2_) were purchased from Concord Technology. ^13^CO_2_ (^13^C, 99%; ^18^O, <2%) was purchased from Cambridge Isotope Laboratories. All the chemicals were analytical grade and used without further purification. Deionized water was prepared with a Milli-Q purification system and used throughout all the experiments.

**Synthesis of TiO_2_/MFU-4l heterojunction**

TiO_2_/MFU-4l heterojunction composite material was synthesized by hydrothermal method. 1g ZnCl_2_, 100 mg H_2_BTDD, and a certain amount of TiO_2_ (P25) were added to 60 mL DMF, sonicated for 30 minutes, and then transferred into the Teflon-lined autoclave and heated at 140 ℃ for 18 h, and then naturally cooled to room temperature. The precipitate was collected by centrifuged and washed with DMF, MeOH, and CH_2_Cl_2_. Then the solid was dried at 80 ℃ under vacuum overnight. The theoretical mass ratios of added TiO_2_ to MFU-4l are specifically 0.42, 0.83, 1.25, 1.67, 2.08, and 2.5. The mass ratio of added TiO_2_ to MFU-4l is 1.25 without specific explanation. MFU-4l was also synthesized using a similar method but without the addition of TiO_2_.

**Synthesis of Au/TiO_2_/MFU-4l**

Au nanoparticles were deposited on TiO_2_/MFU-4l using photo-reduction method. Typically, 50mg of TiO_2_/MFU-4l is dispersed in 200 ul of triethylamine and 50 ml of water. Subsequently, different amounts of HAuCl_4_ solution (10.0 g/L) are added to the suspension solution and sonicated for 10 minutes to promote material dispersion. The reaction mixture was then degassed with argon to remove oxygen for 30 min. Then illuminate the suspended solution under a mercury lamp (300 W) for 60 minutes. Finally, wash the obtained sample with deionized water and dry it in a vacuum oven at 80° C for 24 h. The obtained samples are represented as x% Au/TiO_2_/MFU-4l, where x=12.0, 8.0, 6.4, 4.0, and 1.6 correspond to different amounts of Au added. The content of Au added is 8% without specific explanation. Au/TiO_2_ was also synthesized using a similar method.

**Synthesis of Au@TiO_2_/MFU-4l**

Au@TiO_2_/MFU-4l was obtained by physically mixing Au nanoparticles on TiO_2_/MFU-4l as follows: 1 ml of 1% HAuCl4 solution was added to 95 ml of distilled water and stirred at 130 ° C for 20 minutes. Then, 1 ml of 1% sodium citrate water solution was added and stirred for another 10 minutes. After cooling, a gold colloidal solution was obtained. A certain amount of TiO_2_/MFU-4l was mixed with the gold colloidal solution at room temperature for one hour and centrifuged and washed to obtain Au@TiO_2_/MFU-4l.

**Synthesis of Au/TiO_2_/Cu-MFU-4l, Au/TiO_2_/Ni-MFU-4l, Au/TiO_2_/Cd-MFU-4l**

Following the previously reported method,^[1]^ copper(II) chloride (8.15 mmol) and TiO_2_/MFU-4l (0.2 g) were dissolved in DMF (35 ml), and the reaction mixture was heated at 60 °C for 20 hours. N, N-dimethylacetamide was exchanged with fresh N,N-dimethylacetamide, and then added copper(II) chloride (5.6 mmol) and heated at 60 °C for 20 hours. The precipitate was collected by centrifuged and washed with DMF, MeOH, and CH_2_Cl_2_. Then the solid was dried at 80 °C under vacuum overnight to obtain TiO_2_/Cu-MFU-4l. Using a similar synthesis method, TiO_2_/Ni-MFU-4l and TiO_2_/Cd-MFU-4l were obtained by replacing copper (II) chloride with nickel (II) chloride and cadmium (II) chloride, and then gold nanoparticles were loaded by photoreduction.

**Characterization**

Scanning electron microscope (SEM) images were taken on a HITACHI S-4800 microscope. Transmission electron microscopy (TEM) images and energy dispersive X-ray (EDX) analysis elemental maps were taken on a Hitachi JEM-2100F microscope. Powder X-ray diffraction (PXRD) patterns of the catalysts were analyzed by using a Bruker D8-advance with CuKα radiation (λ = 1.5406 Å), which operated at 40 kV voltage and 40 mA current. X-ray photoelectron spectroscopy (XPS) was conducted on a PHI Quantera SXM X-ray photoelectron spectrometer equipped with an Al X-ray excitation source (1486.6 eV). All binding energies were calibrated against the C1s peak at 284.8 eV. The UV-vis diffuse-reflection spectra (DRS) were recorded on a UV-vis spectrophotometer (UV-3900) at ambient temperature and pressure. Fourier transform infrared (FT-IR) spectroscopy was performed on a Bruker Vertex 70 V spectrometer equipped with a narrow band HgCdTe detector. Metal content was quantified by inductively coupled plasma optical emission spectra (Agilent 5800 VDV ICP-OES). Photoluminescence spectra (PL) were measured on a fluorescence spectrophotometer (Hitachi F4600) for carriers’ separation of samples. The time-resolved transient PL decay was acquired by FLS-1000 (Edinburgh Instruments). X-ray absorption near-edge structure (XANES) and extended X-ray absorption fine structure spectrum (EXAFS) data were analyzed by Artemis software. TPD measurements were carried out on quantachrome autosorb-iQC chemisorption analyzer with a thermal conductivity detector. Thermogravimetric analyses (TGA) were measured on TG-DTA6300 thermo-analyzer under air atmosphere for all samples. N_2_ and CO_2_ adsorption isotherms were collected on an ASAP 2020HD88 apparatus. The Brunauer-Emmett-Teller (BET) surface areas of samples were analyzed by N_2_-adsorption-desorption isotherms at 77 K and the distribution of pore size was determined according to the BET equilibrium equation. Electron paramagnetic resonance (EPR) spectra were performed by a Bruker Elexsys E500-T spectrometer.

The Mott-Schottky analysis was conducted on an electrochemical workstation (Autolab, PGSTAT 302 N, Metrohm) in a standard three-electrode one-compartment (20 mL) configuration with the photocatalyst-coated FTO as the working electrode, Pt wire as the counter electrode, and Ag/AgCl as the reference electrode. Besides, 0.5 M of Na_2_SO_4_ solution was used as the electrolyte solution. Electrochemical test was then conducted at frequencies of 500, 750, and 1000 Hz, respectively. The working electrode was prepared according to the following procedure, 10 mg of the photocatalyst was added to a mixture of Nafion (150 μL) and ethanol (850 μL). Next, the obtained suspension (200 μL) was dropped on the surface of the FTO substrate with a working area of 2 × 2 cm^2^.

**Photocatalytic CO_2_ reduction measurements**

In a typical process, 5 mg of catalyst and 100 μl of TEA were added to 20 mL of water. Then degas the reaction mixture with pure CO_2_ to remove oxygen for at least 30 minutes. A 300 W xenon lamp with an AM1.5 filter (Beijing Perfectlight, Microsolar 300, 350–1000 nm, 300 mW·cm^2^) was used as the light source. The reaction temperature was maintained at 25 °C by a circulation water chiller. During the reaction, 1mL of gaseous products from the reactor were sampled and monitored using a gas chromatography (Fuli GC-2060) equipped with FID and TCD detector, and H_2_, CO, CH_4_, C_2_H_4_, and C_2_H_4_ were well-resolved. Liquid products (methanol, ethanol) were monitored using gas chromatography-mass spectrometry (GCMS-QP2010). Evacuate the reaction gas through the bubbling method and introduce new carbon dioxide gas for cyclic experiments, and replenish the sacrificial agent every two cycles. At the same time, blank experiments were conducted under the conditions of introducing argon gas and no light to determine that the product of the reaction came from photocatalytic carbon dioxide reduction. ^13^CO_2_ isotope labeling experiments were conducted under the same conditions, and the gas products of the isotope experiments were analyzed using Agilent gas chromatography-mass spectrometer (Agilent Technologies 8890B-5977B).

**Results and Discussion**

**Structural analysis**

As shown in Figure 2a-e, the Ti 2p spectrum of TiO_2_ shows two peaks at 458.6 eV and 464.3 eV, corresponding to Ti 2p3/2 and Ti 2p1/2, respectively.^[2]^ The O1s spectrum shows Ti-O lattice oxygen at 529.7 eV. The Zn 2p spectrum of MFU-4l shows two peaks at 1022.45 eV and 1045.45 eV, corresponding to Zn 2p3/2 and Zn 2p1/2, respectively. In addition, MFU-4l shows a broad peak at 198 eV, corresponding to the binding energy of 2p electrons in Cl, indicating that Zn^2+^ in the tetrahedral coordination structure coordinates with three BTDD^2-^ ligands and one Cl^-^ ion.^[1]^ After the formation of TiO_2_/MFU-4l composite material, the binding energy of Zn 3p shifts negatively by 0.8 eV, the binding energy of Ti 2p remains unchanged, and the binding energy of O1s in Ti-O shifts positively by 0.1 eV, indicating electron migration from TiO_2_ to MFU-4l.

Since MFU-4l moiety has two Zn^2+^ sites (N_3_ZnCl and N_6_Zn) with different coordination environments, the result of fitting EXAFS is an average coordination number.^[3,4]^ According to our fitting results (Figure S6-S7 and Table S2), the Zn-O/N and Zn-Cl coordination numbers of Zn^2+^ in pure MFU-4l are 4.5 and 0.9, respectively. When TiO_2_ is loaded, the Zn-Cl of Zn^2+^ in TiO_2_/ MFU-4l disappears and the Zn-O/N coordination number increases to 4.9, which is attributed to the oxygen on the hydroxyl group on the surface of TiO_2_ replacing the Cl in the tetracoordinated N_3_ZnCl and forming the N_3_ZnO structure. Wavelet transform (WT) analysis provided high-resolution identification in both K space and R space (Figure S8). It can be analyzed that the highest intensity peaks of 1.4 and 1.7 Å in R space correspond to Zn-O/N and Zn-Cl coordination, respectively. Indeed, the WT spectrum of TiO_2_/MFU-4l detected a maximum peak intensity of 1.4 Å in the R space, indicating that only Zn-N/O coordination exists. Furthermore, we conducted spectral analysis of the Au L3 edge XANES and EXAFS to determine the coordination environment of Au in the Au/TiO_2_/MFU-4l catalyst (Figure 2h and S9).^[5]^ From the EXAFS analysis and WT spectra (Figure S10), the peaks observed at 2.5 Å and 1.6 Å for the Au/TiO_2_/MFU-4l catalyst are attributed to Au-Au and Au-N/O, respectively. According to the results of the fitting analysis (Table S2), the coordination number of Au-O is close to 2, and the coordination number of Au-Au is close to 8, indicating that the Au element mainly exists in the form of nanoparticles, but there is still a portion of Au-O bonds.

**The band-structure characterization**

The band structures of the sample were measured by UV visible diffuse reflectance spectroscopy (UV visible DRS spectroscopy) and Mott Schottky (M-S). From the Tauc plots (Figure S19), the band gaps of TiO_2_ and MFU-4l were calculated to be 3.40 and 3.36 eV, respectively. From the positive slope of the M-S plot (Figure S20), it can be seen that TiO_2_ and MFU-4l are both n-type semiconductors. In addition, according to the M-S plot, the flat band potentials of TiO_2_ and MFU-4l were calculated to be -0.73 V and -1.08 V (vs. Ag/AgCl), respectively. For n-type semiconductors, the flat band potential is close to the conduction band (CB). According to the conversion formula E (vs. NHE) = E (vs. Ag/AgCl) +0.1976, the CB of TiO_2_ and MFU-4l can be estimated to be -0.53 V and -0.88 V (vs. NHE), respectively.^[6]^ Based on the CB and band gap obtained above, the valence band (VB) of TiO_2_ and MFU-4l can be calculated to be 2.87V and 2.48V, respectively. Since E_0_ of CO_2_/CO = -0.52 V vs. NHE and CO_2_/C_2_H_4_ = -0.34 V vs. NHE are both lower than the CB of TiO_2_ and MFU-4l, theoretically, Au/TiO_2_/MFU-4l has a suitable band gap to reduce carbon dioxide to CO and C_2_H_4_.^[7]^

**In situ infrared detection of reaction intermediates**

Under light irradiation, the in-situ FT-IR spectra of Au/TiO_2_/MFU-4l showed multiple peaks that increased with irradiation time (Figure 5a). The peaks at 1349 and 1397 cm^-1^ were attributed to the bidentate carbonate (b-CO_3_^2−^) group,^[8]^ while the peaks at 1293 cm^-1^ and 1452 cm^-1^ were related to the monodentate carbonate (m-CO_3_^2−^) group.^[9,10]^ Besides, the peaks at 1612cm^-1^ and 1656cm^-1^ can be attributed to the vibration of the *COOH group.^[11]^ Importantly, the peaks at 1367cm^-1^ and 1477cm^-1^ can be attributed to *COCO, which was generally regarded as the crucial intermediate to form *C_2_H_4_.^[12]^ This indicates the formation of *COCO intermediates on Au/TiO_2_/MFU-4l photocatalysts, confirming the occurrence of the C-C coupling process on the catalyst surface. However, almost no reaction intermediates were detected on pure MFU-4l and TiO_2_ (Figure S22), indicating that the carbon dioxide reduction reaction was difficult to occur on the surface of the two catalysts alone. The signals of these reaction intermediates were also detected on TiO_2_/ MFU-4l, but the signal intensity was weak. In contrast, Au/TiO_2_/MFU-4l photocatalyst showed the most obvious IR signals in the shortest illumination time, indicating its highest photocatalytic activity for carbon dioxide reduction.

**The adsorption and binding ability of catalysts for gases**

The adsorption and activation of CO_2_ is one of the important processes in the entire reaction. We further validated the crucial role of MFU-4l in gas adsorption and binding during the reaction process. From CO_2_ adsorption isotherms (Figure S23), due to the porous structure of MFU-4l, it shows the highest amount of CO_2_ adsorption capacity (19.6 cm^3^/g). Compared with pure TiO_2_, the CO_2_ adsorption capacity of TiO_2_/MFU-4l (8.9 cm^3^/g) and Au/TiO_2_/MFU-4l (6.0 cm^3^/g) is significantly improved, which is more conducive to the surface uptake of CO_2_ by the catalyst. The adsorption and desorption abilities of these catalysts towards the reaction gas CO_2_, key intermediate CO, and main product C_2_H_4_ were further evaluated through temperature-programmed desorption measurements. As shown in Figure 3c-e, pure MFU-4l exhibits strong binding ability to three gases due to its excellent specific surface area and pore structure. Pure TiO_2_ has a weak binding ability to CO_2_ and CO, and a strong binding ability to C_2_H_4_, which is not conducive to the reaction. Compared with pure TiO_2_, the introduction of MFU-4l significantly improves the chemisorption ability of TiO_2_/MFU-4l and Au/TiO_2_/MFU-4l for CO_2_ and CO, which is very beneficial for the C-C coupling process. Especially, the experimental results of C_2_H_4_ temperature programmed desorption (C_2_H_4_-TPD) showed that the deposition of Au NPs by photo-deposition can reduce the C_2_H_4_ adsorption strength of the catalyst. Compared with the other materials, Au/TiO_2_/MFU-4l showed the weakest C_2_H_4_ affinity, significantly improving the selectivity of C_2_H_4_. Therefore, Au/TiO_2_/MFU-4l material exhibits good ability in adsorbing reaction gas CO_2_, enriching C-C coupling intermediate CO, and dissociating product C_2_H_4_, which is very beneficial for photocatalytic reduction of CO_2_ to produce C_2_H_4_. In addition, we also measured the H_2_-TPD profiles of different materials (Figure S24). The binding ability of the samples containing MFU-4l to hydrogen is also stronger than that of pure TiO_2_, which may be one of the reasons why Au/TiO_2_/MFU-4l can effectively inhibit the competitive reaction of hydrogen evolution in the photocatalytic reduction of carbon dioxide.


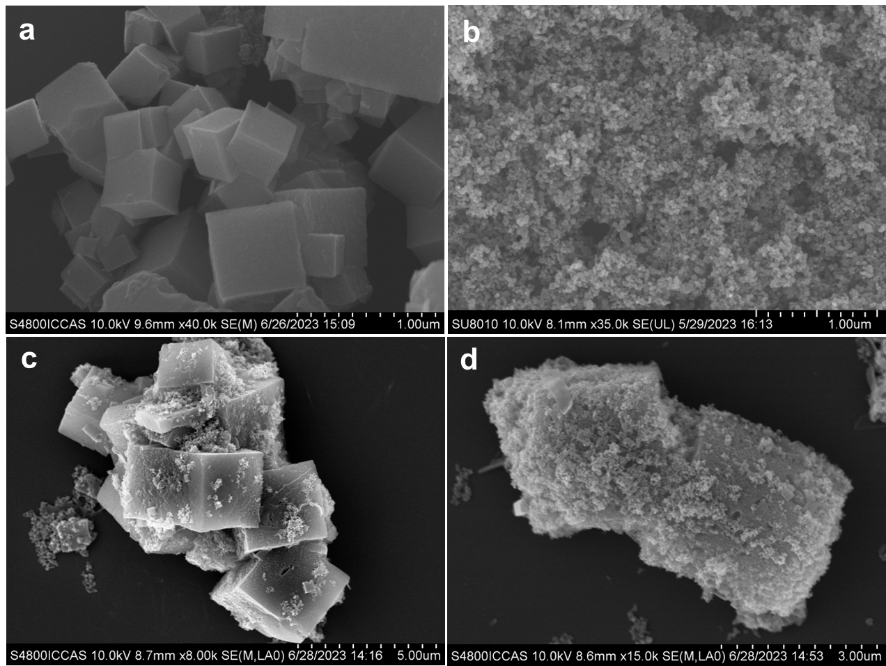


**Figure S1.** SEM images of (a) MFU-4l, (b) TiO_2_, (c) TiO_2_/MFU-4l, and (d) Au/TiO_2_/MFU-4l.


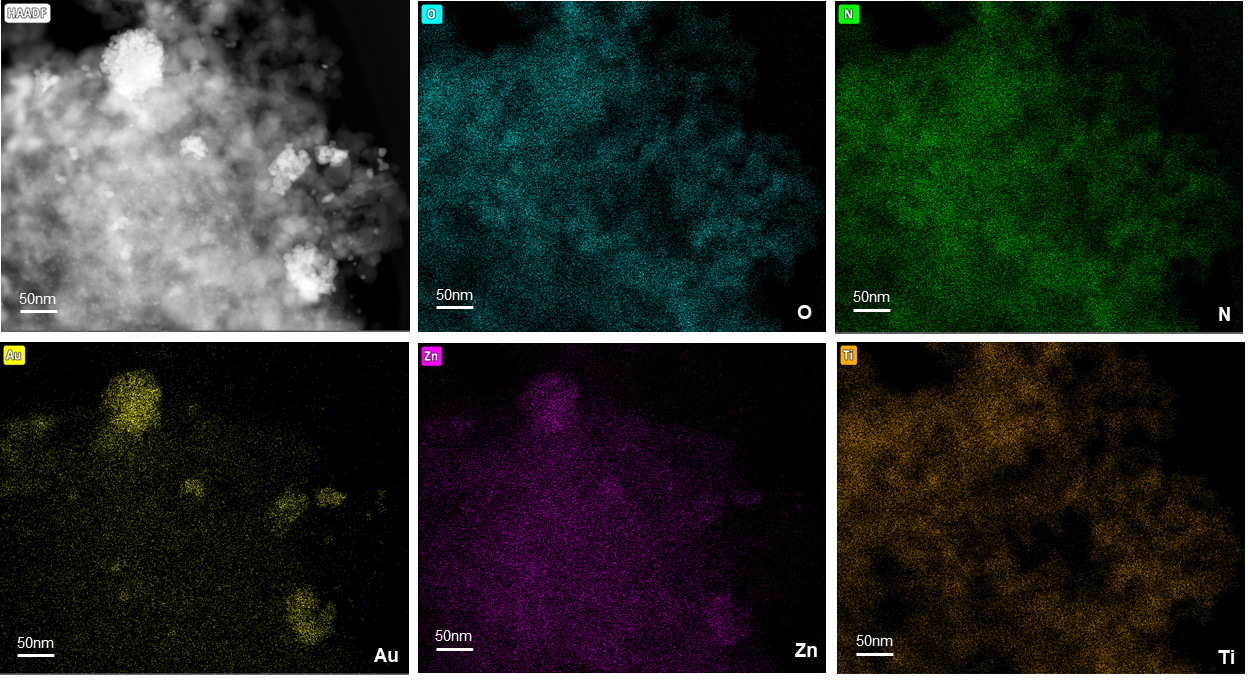


**Figure S2.** High-angle annular dark-field (HAADF) and EDX elemental mapping images of Au/TiO_2_/MFU-4l.


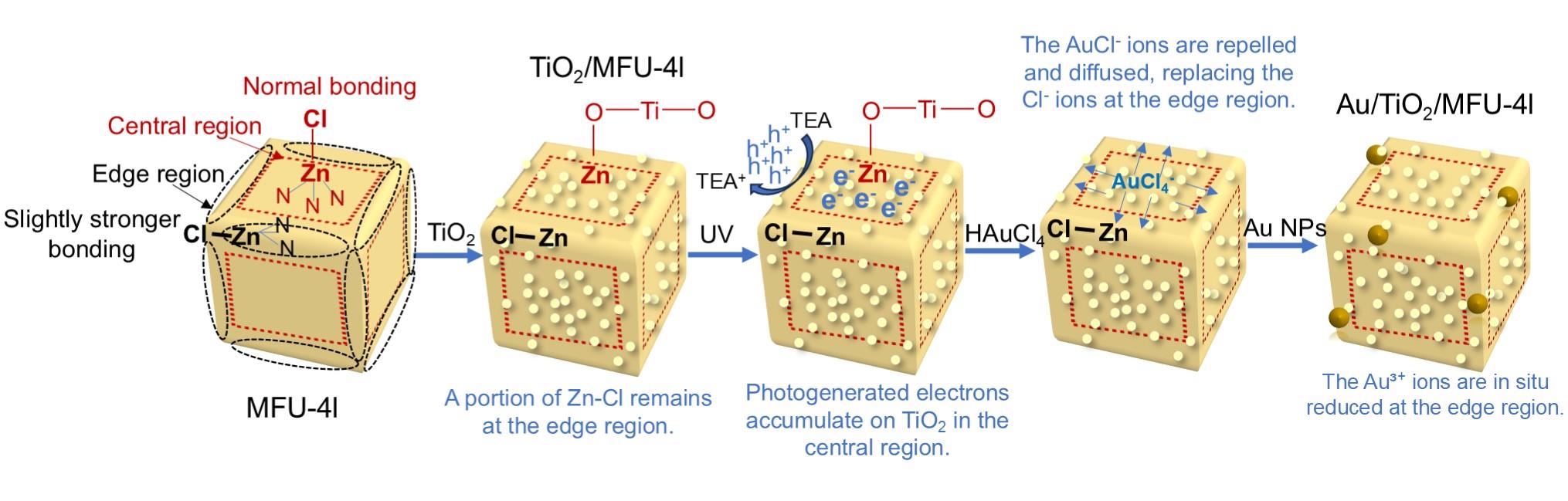


**Figure S3.** Illustration of the deposition of Au nanoparticles (NPs) on the edge region of TiO_2_/MFU-4l.

Before photo-deposition of AuCl_4_^-^, as shown in Figure 2c, most of Cl^-^ ligands at the apex of the tetrahedra within the nodes of MFU-4l have been substituted by suspended Ti-O^-^ bonds of TiO_2_ surface when the TiO_2_/MFU-4l heterojunction was prepared by the hydrothermal method. However, there were still some remaining Cl^-^ sites that were not easily replaced (refer to the schematic Figure S3). And these Cl^-^ sites were most likely located at the edge positions of the quasi-cubes, where the zinc ions attached to them are highly unsaturated (due to the disruption of the periodic structure here) and exhibit stronger Zn-Cl bonding relative to that of the quasi-cube's flat surface body. Upon UV light irradiation, the photo-induced electrons of the TiO_2_/MFU-*4l* photocatalyst were readily trapped on TiO_2_ sites located in the central region of the quasi-cube, which resulted in the center region being more negatively charged. When the AuCl_4_^-^ solution was added to this photo-deposition system, the negatively charged AuCl_4_^-^ ions were forced to diffuse or migrate toward the remaining Cl^-^ sites at the periphery of the quasi-cube of TiO_2_/MFU-4l and substituted these remaining Cl^-^ sites. Subsequently, these AuCl_4_^-^ ions were photo-reduced in situ into Au-clusters or Au nanoparticles at the edge region of the TiO_2_/MFU-4l structure, as confirmed by the HAADF images of Figure 1e. As shown in Figure 2c, no Cl^-^ ligands remained after the photo-deposition of Au nanoparticles.


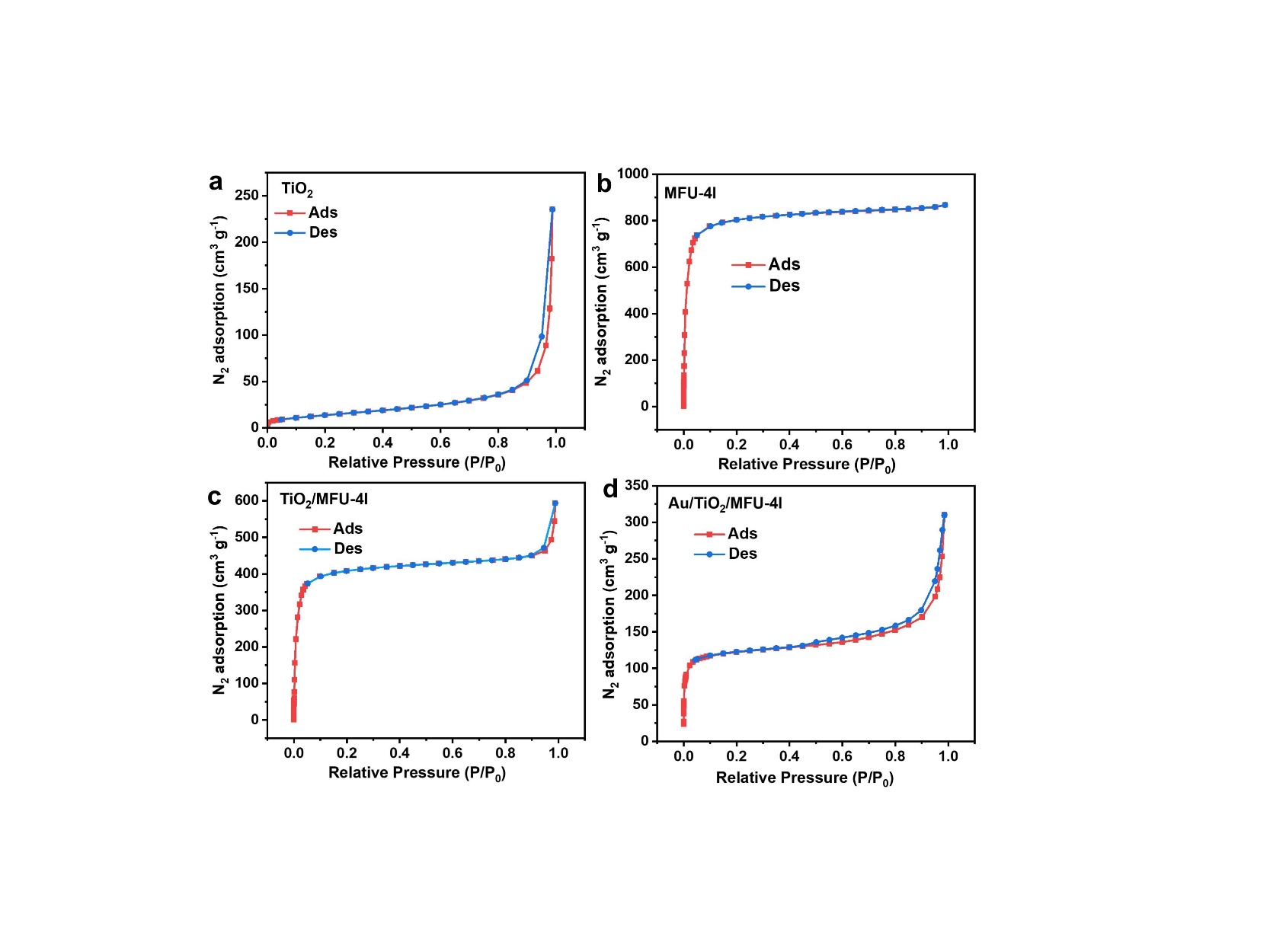


**Figure S4.** N_2_ adsorption/desorption isotherms.


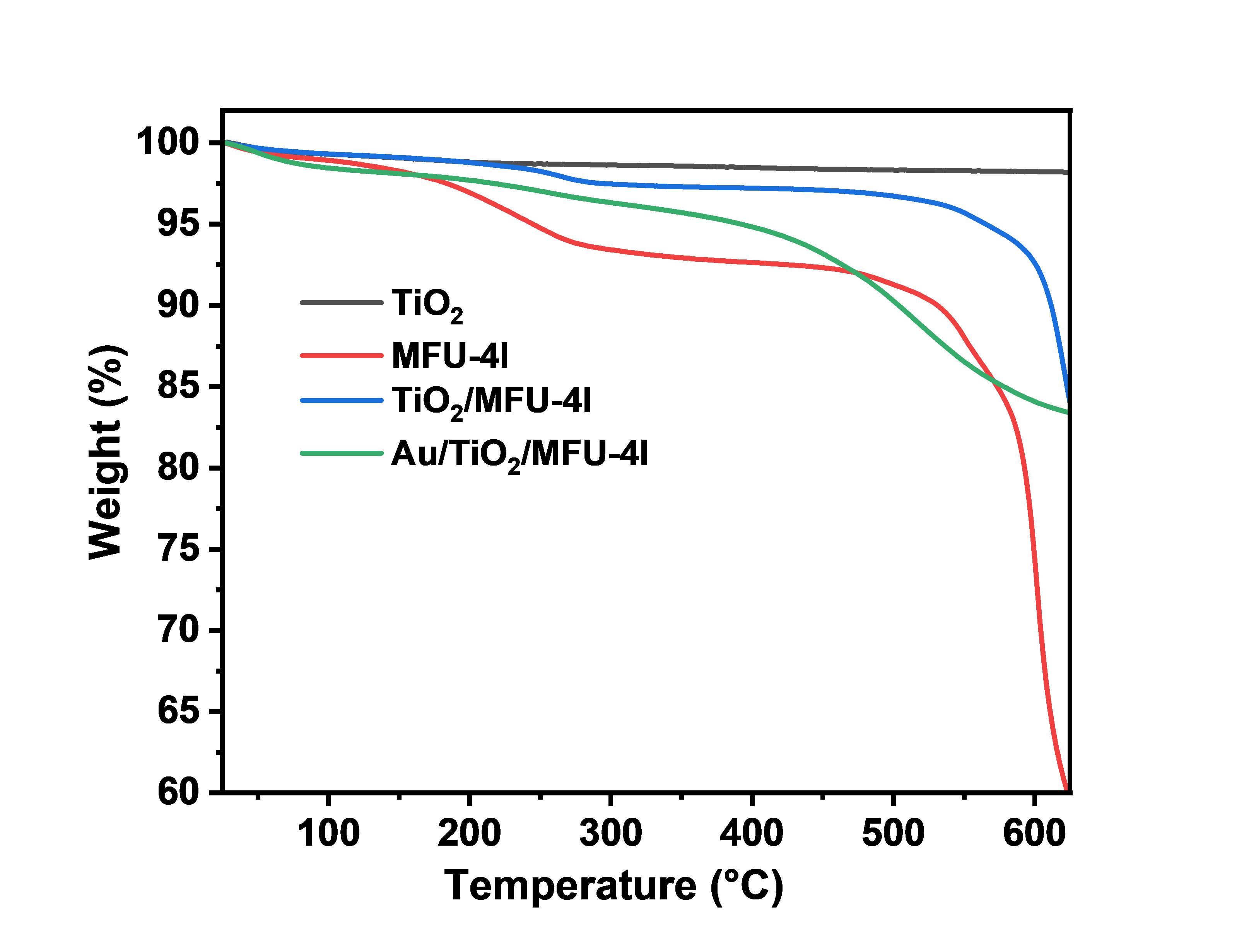


**Figure S5.** TGA of different samples.

**Figure S6**. Zn K edge FT-EXAFS spectra.


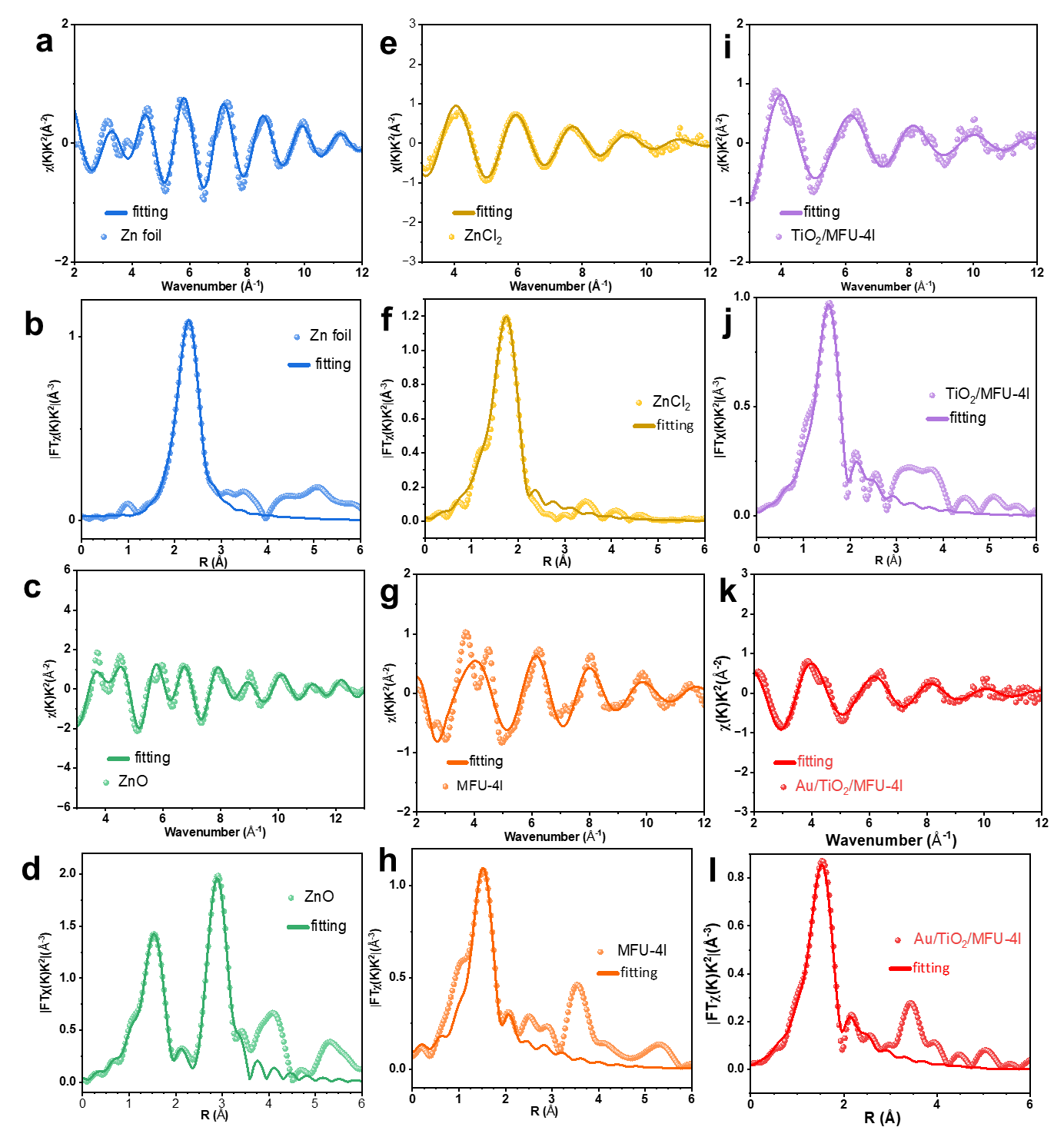


**Figure S7**. Fitting of the EXAFS spectrum of Zn foil (a, b), ZnO (c, d), ZnCl_2_ (e, f), MFU-4l (g, h), TiO_2_/MFU-4l (i, j) and Au/TiO_2_/MFU-4l (k, l) in k space and R space.


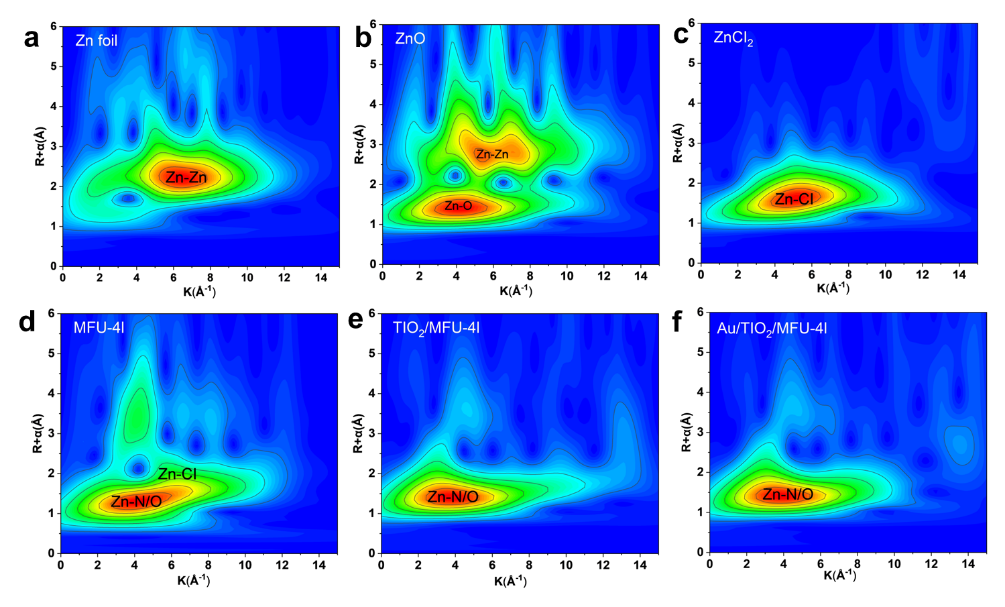


**Figure S8.** WT-EXAFS of Zn foil (a), ZnO (b), ZnCl_2_ (c), MFU-4l (d), TiO_2_/MFU-4l (e) and Au/TiO_2_/MFU-4l(f).


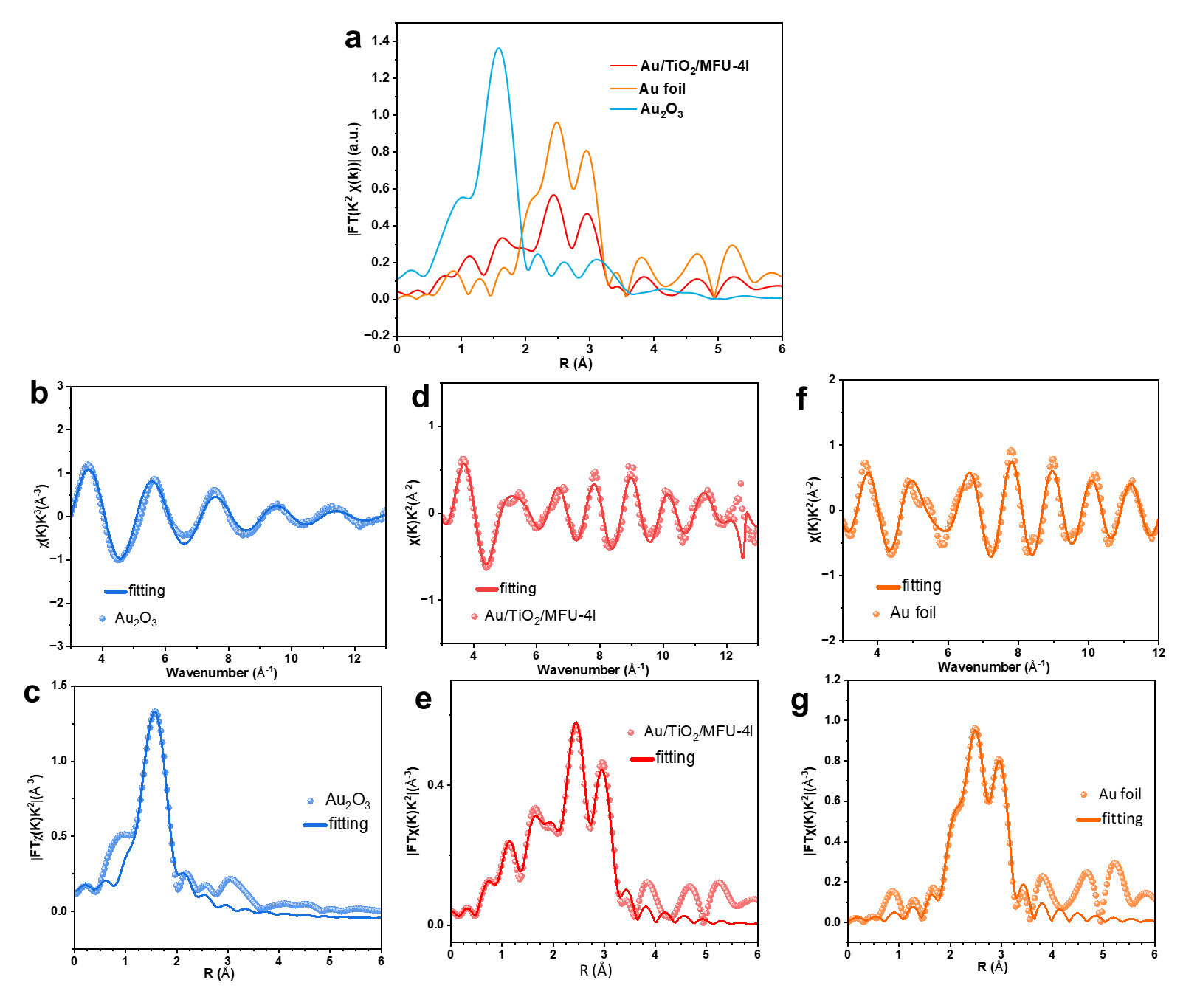


**Figure S9.** (a) Au L3 edge FT-EXAFS spectra and fitting of the EXAFS spectrum of Au_2_O_3_ (b, c), Au/TiO_2_/MFU-4l (d, e), and Au foil (f, g).


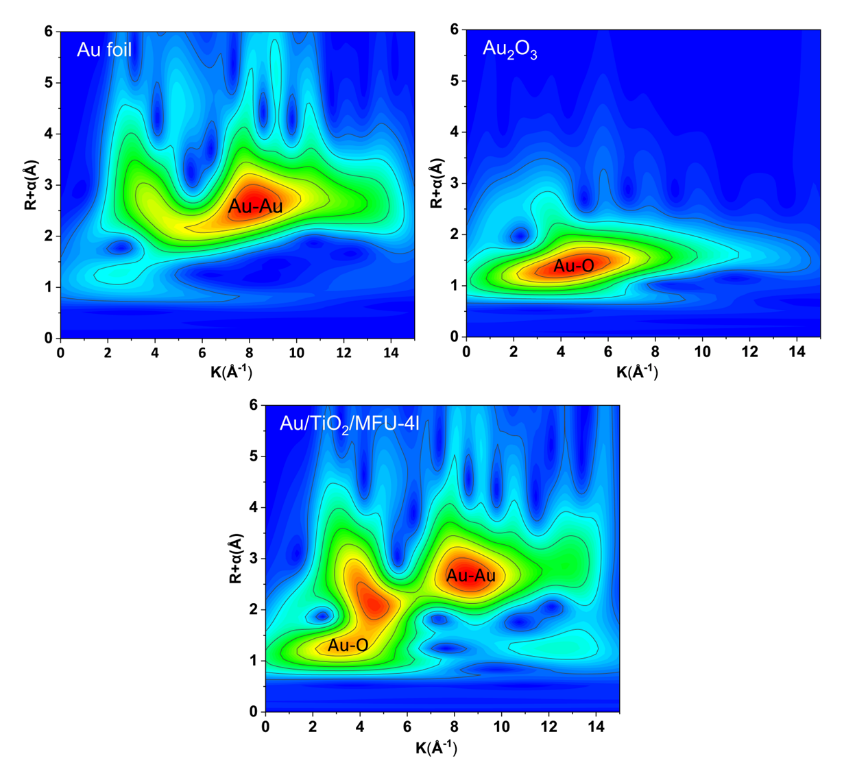


**Figure S10**. WT-EXAFS of Au foil, Au_2_O_3_, and AuTiO_2_/MFU-4l.


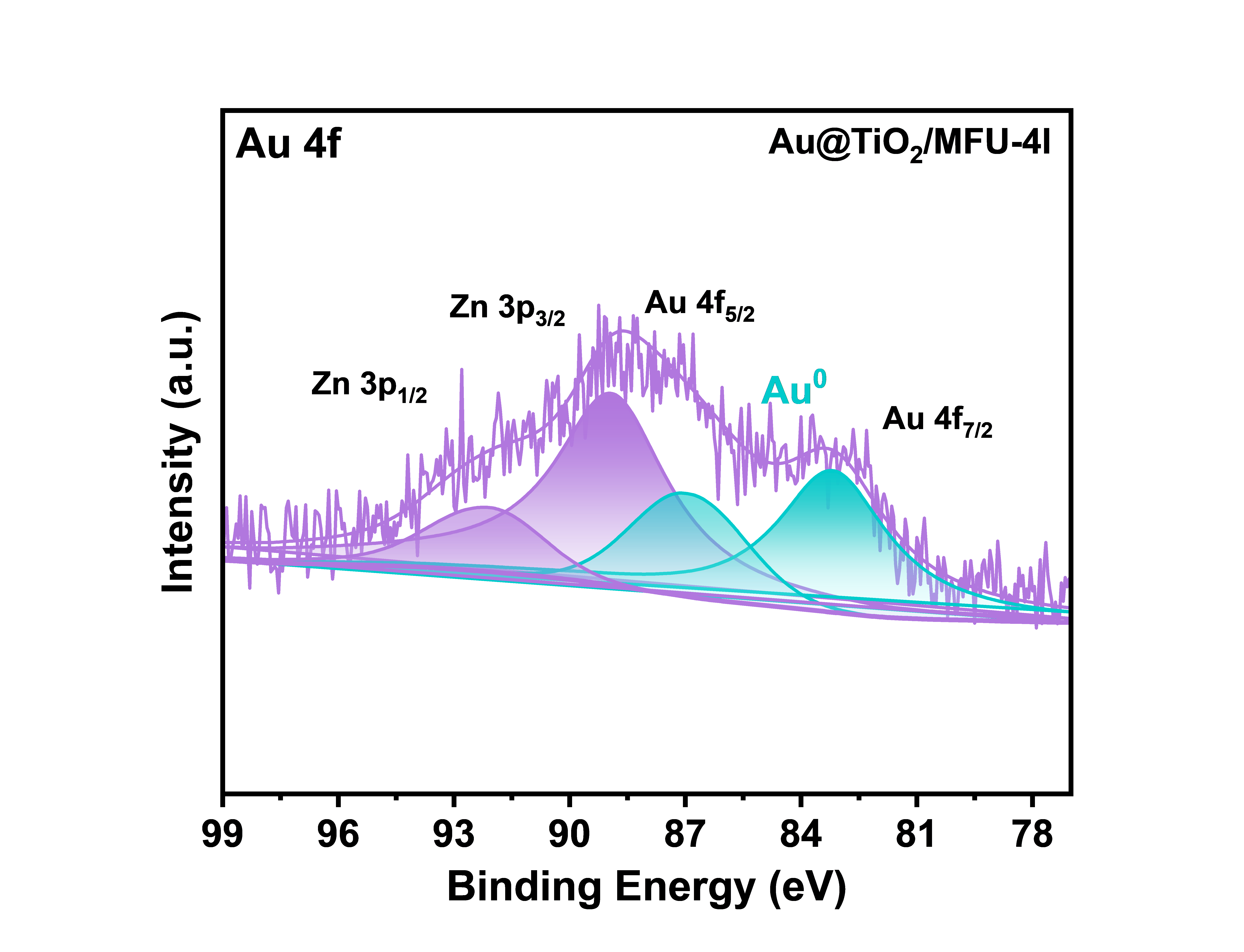


**Figure S11**. XPS spectra of Au 4f of Au@TiO_2_/MFU-4l.


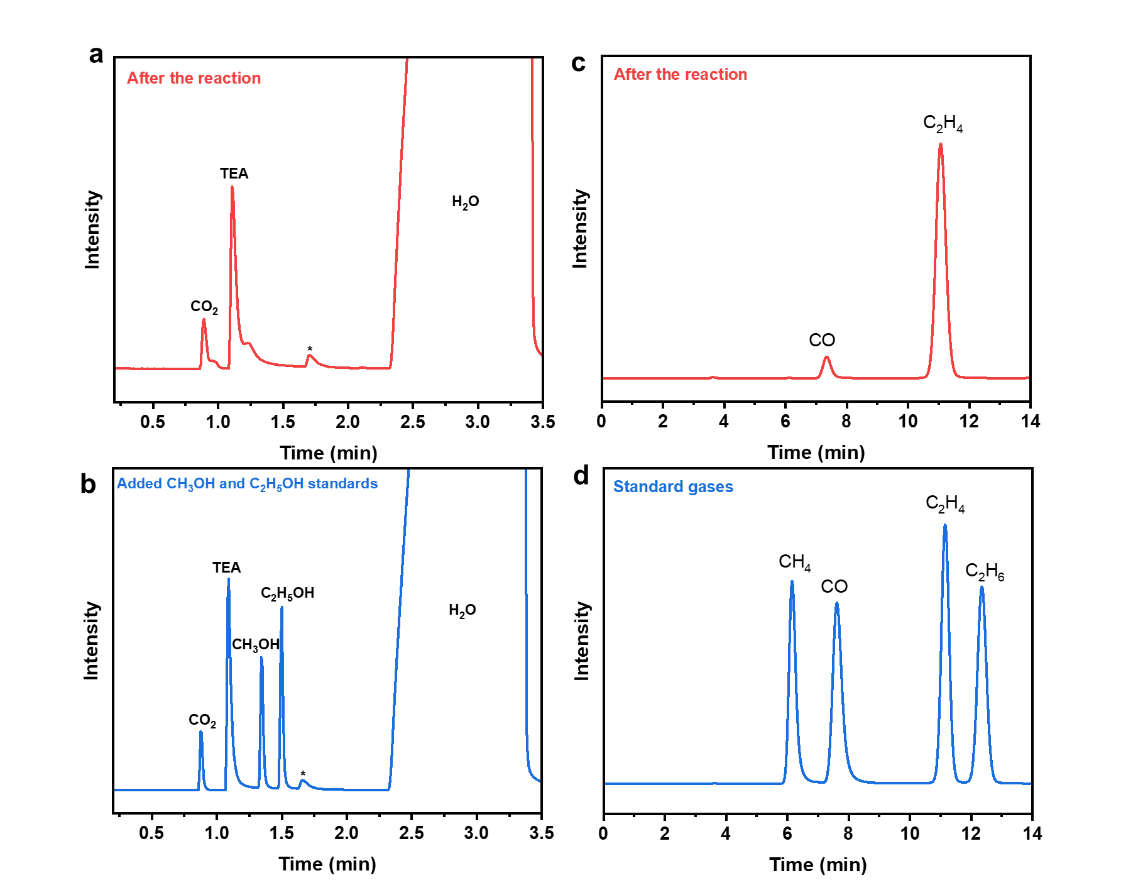


**Figure S12**. (a) GC-MS chromatogram of the liquid products after the reaction were monitored by GC-MS (GCMS-QP2010). (b) GC-MS chromatogram of the liquid sample after the addition of methanol and ethanol standards as references. (c) GC chromatogram of gas products after the reaction were monitored by GC (Fuli GC-2060). (d) GC chromatogram of standard gases used as references.


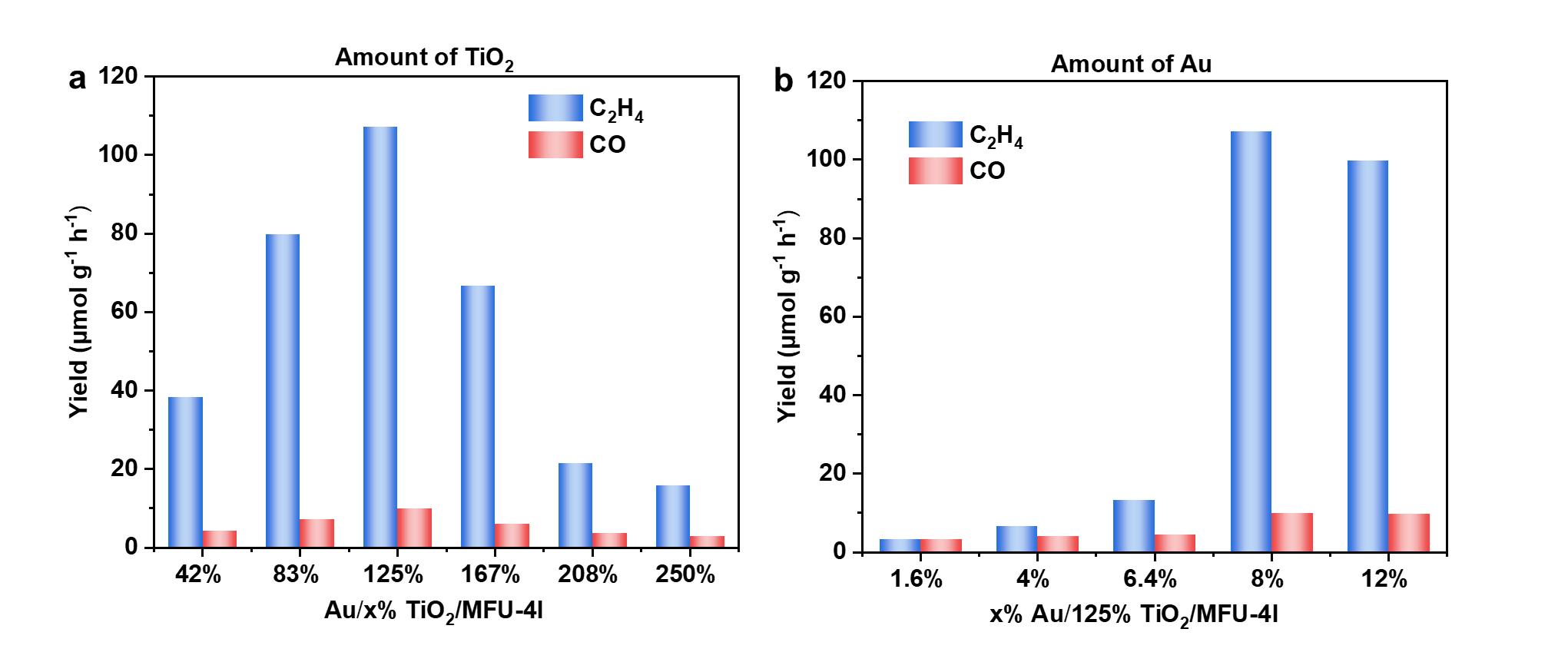


**Figure S13**. Yield of products in photocatalytic CO_2_ reduction reaction of Au/TiO_2_/MFU-4l with different relative contents (wt%) of TiO_2_ (a) and Au nanoparticles (b) to MFU-4l main structure.


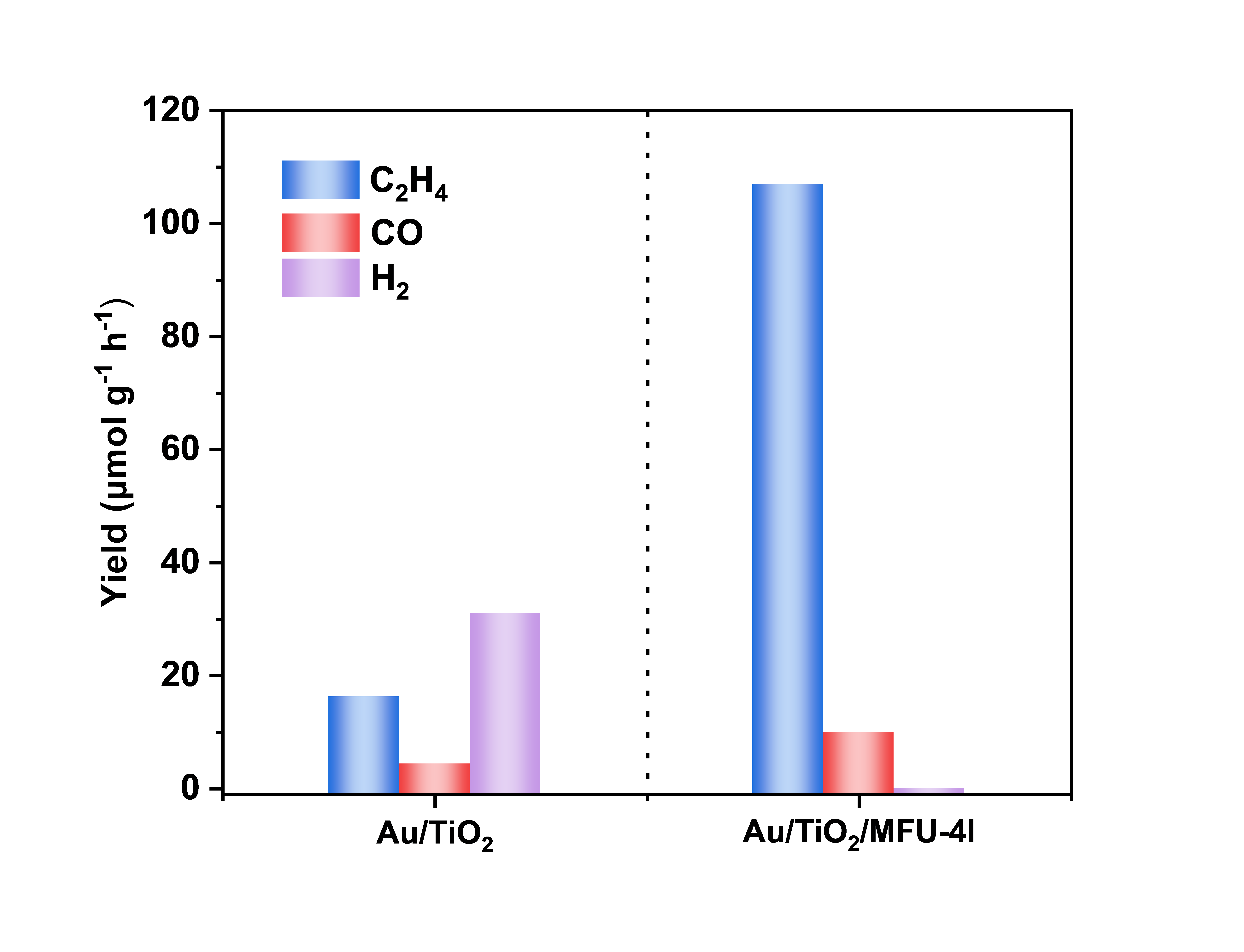


**Figure S14.** Comparison of photocatalytic carbon dioxide reduction and H_2_ evolution reactions with different materials.


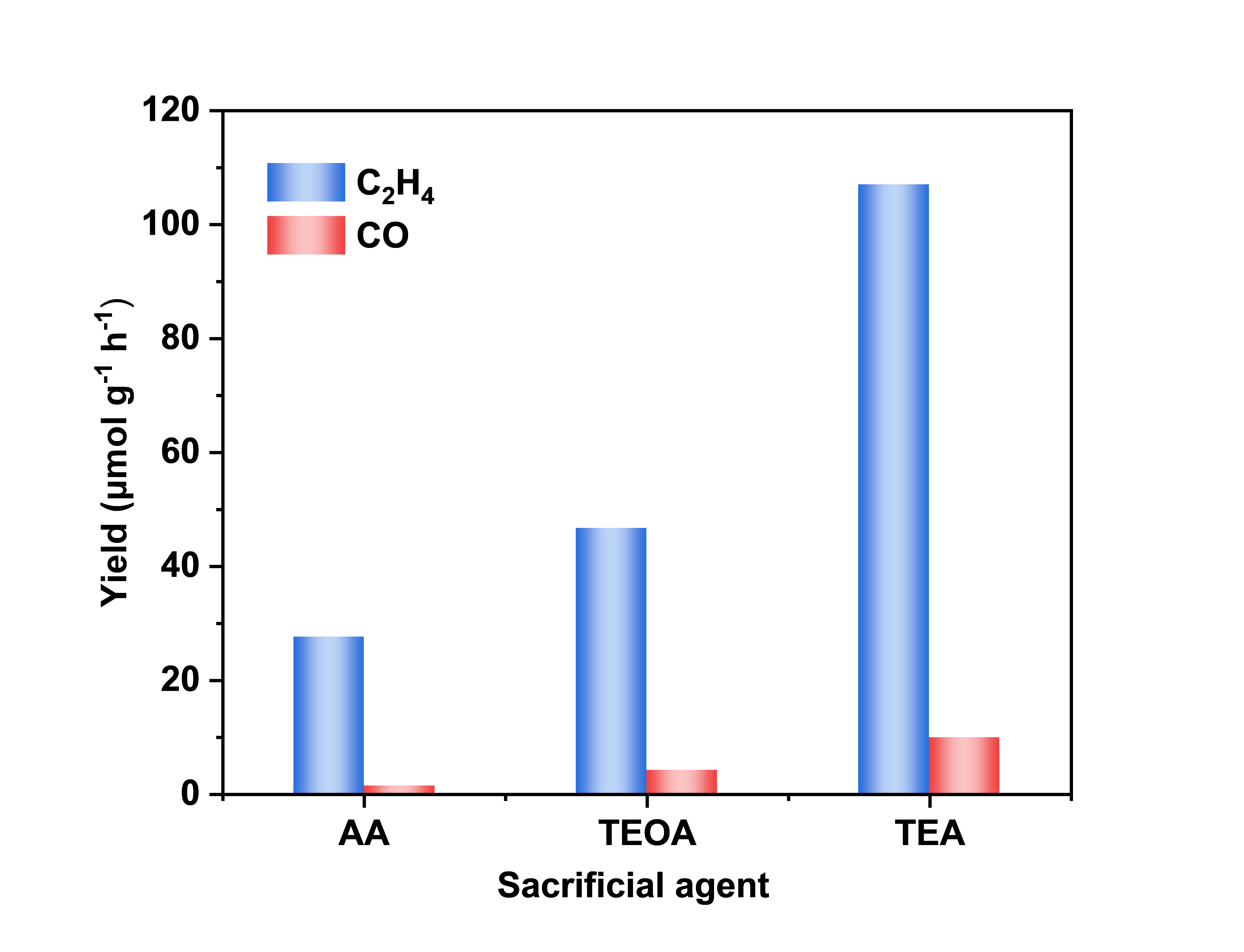


**Figure S15**. Performance tests under different sacrificial agents.


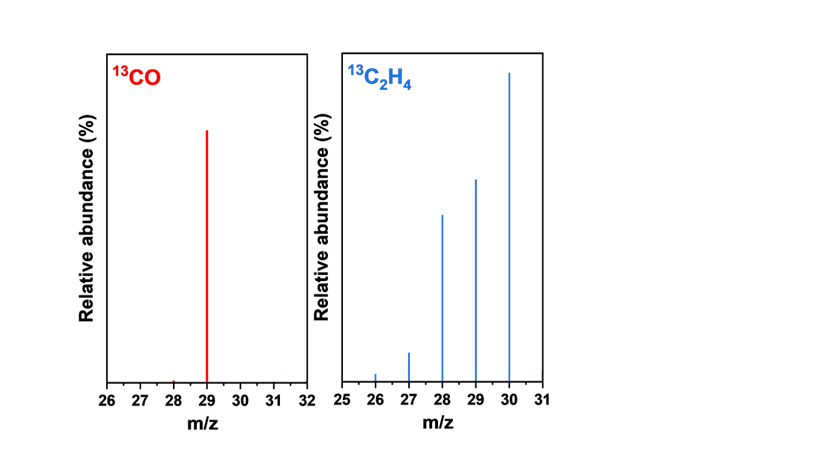


**Figure S16.** GC-MS spectra of CO and C_2_H_4_ from the photocatalytic reduction of ^13^CO_2_.

**Figure S17.** Stability tests (Evacuate the reaction gas and replenish new carbon dioxide gas during each cycle).


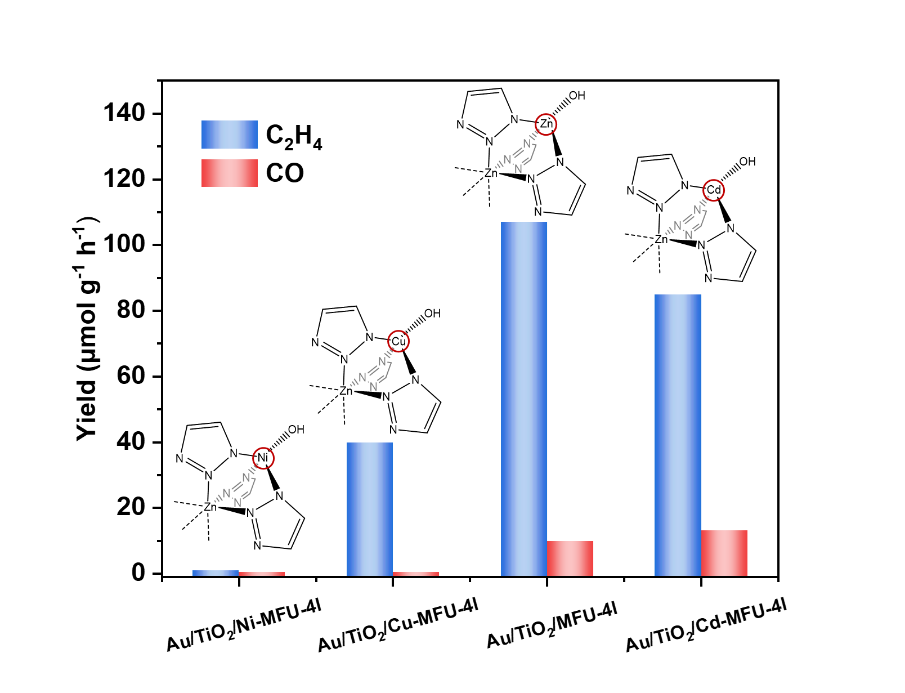


**Figure S18.** Performance tests of four-coordination central metal ion Zn^2+^ partially substituted by other metal ions (Cu^2+^, Ni^2+^, Cd^2+^) in MFU-4l.


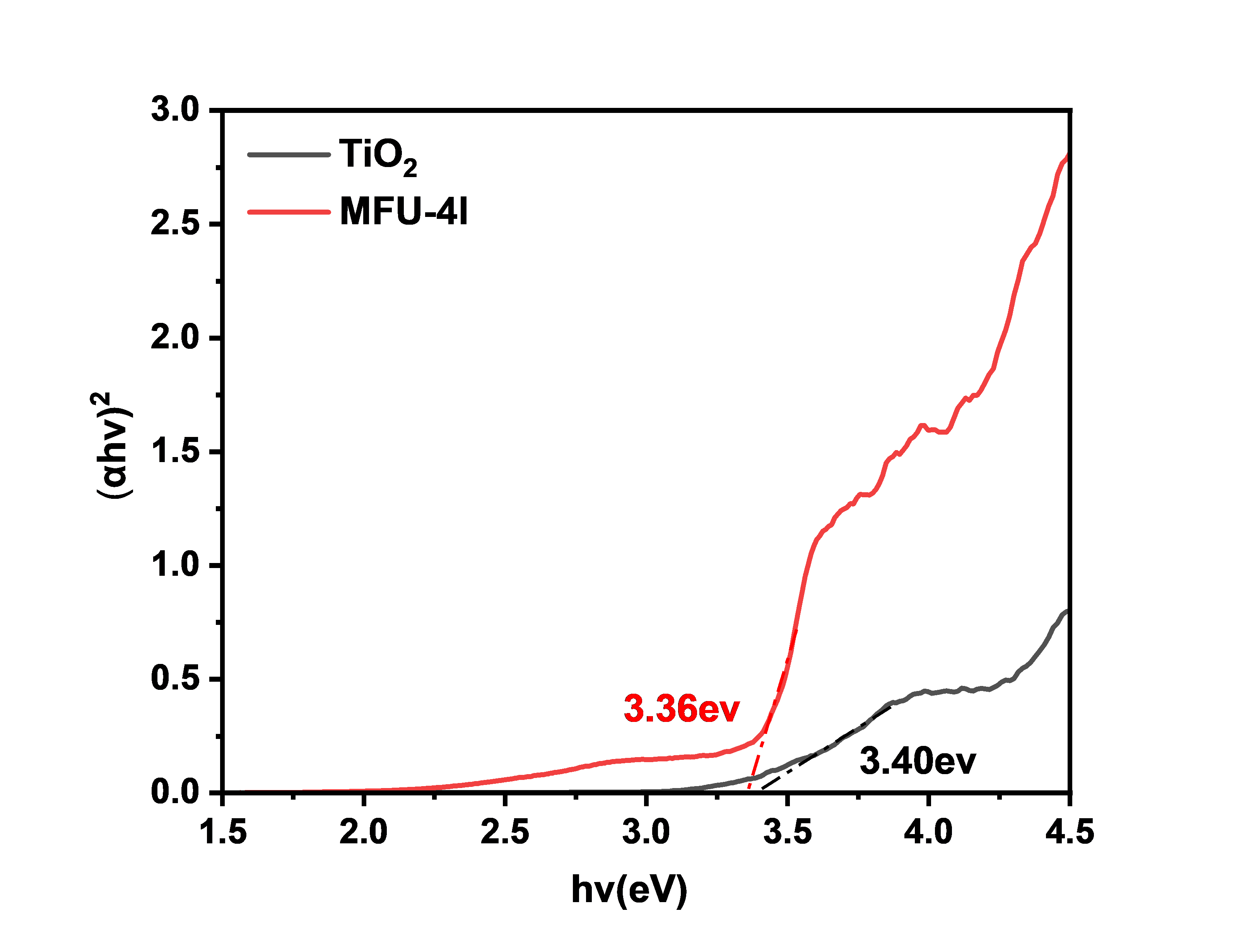


**Figure S19.** Tauc plots.


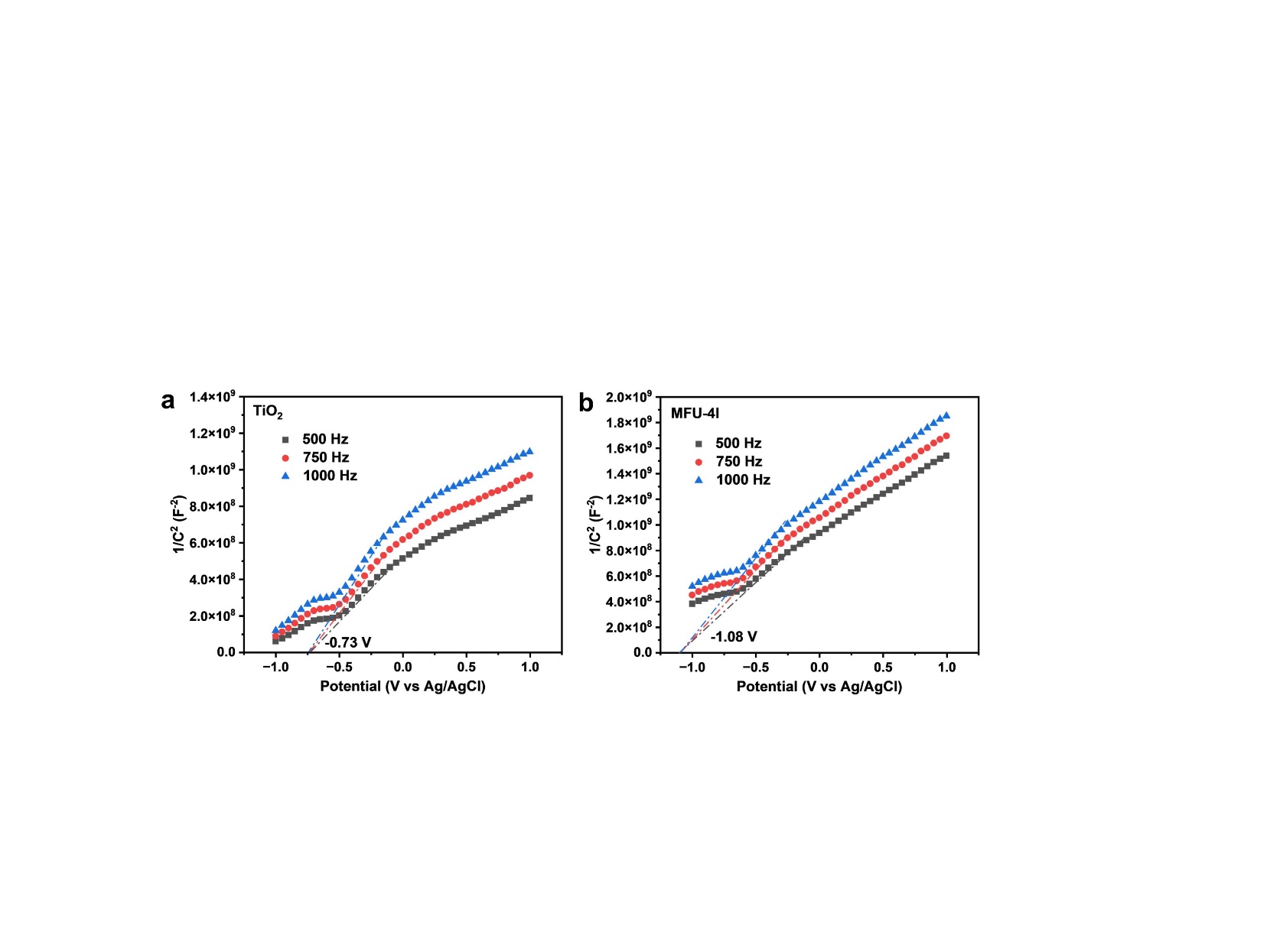


**Figure S20.** M-S plots of TiO_2_ (a) and MFU-4l (b).


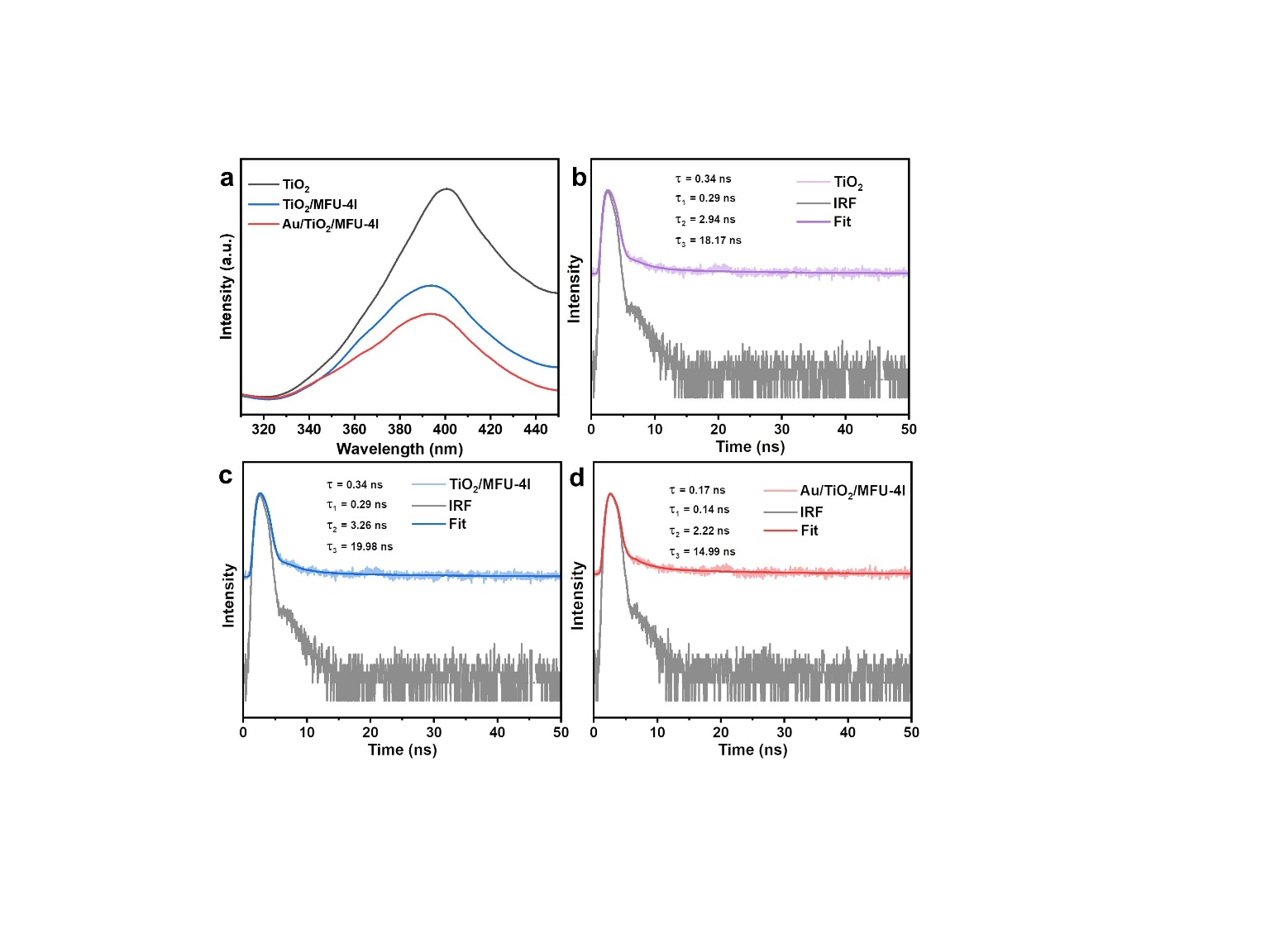


**Figure S21.** (a) PL spectra under an excitation light of 250 nm and time-resolved photoluminescence spectroscopy of (b) TiO_2_, (c) TiO_2_/MFU-4l, and (d) Au/TiO_2_/MFU-4l.

**
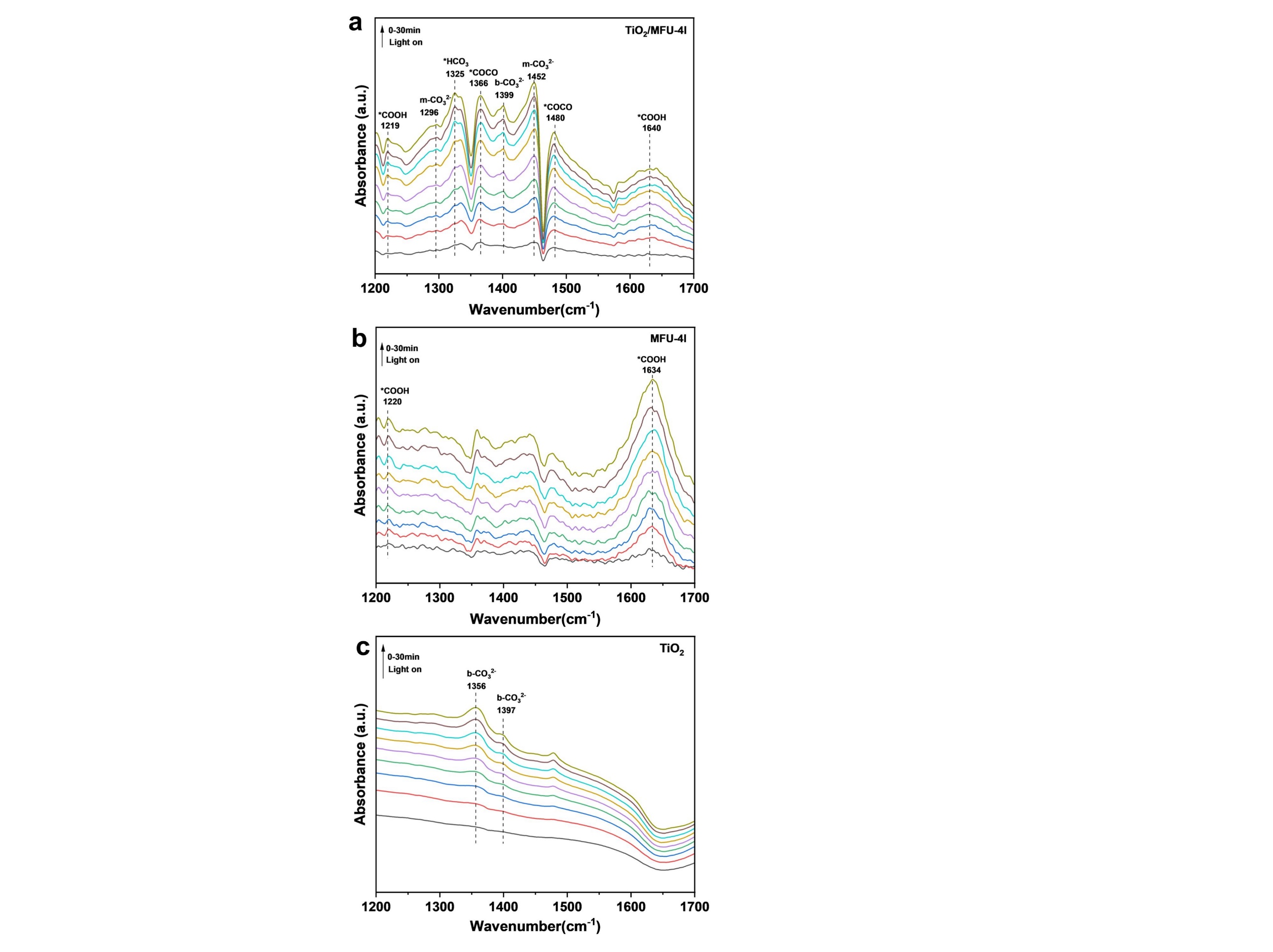
**

**Figure S22.** In situ FTIR spectra on the (a) TiO_2_/MFU-4l, (b)MFU-4l and (c) TiO_2_ for CO_2_ photoreduction process under irradiation.


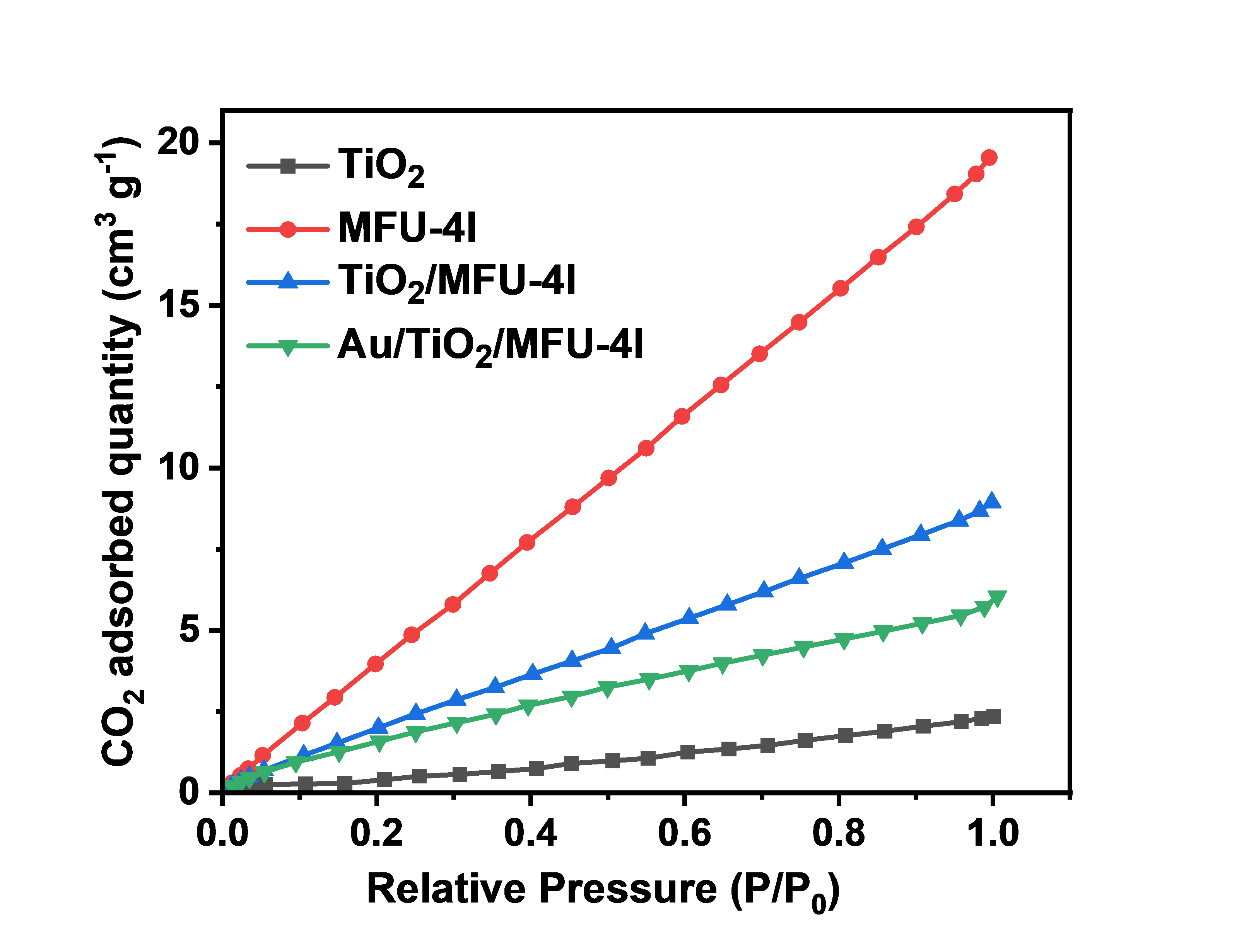


**Figure S23**. CO_2_ adsorption performance.


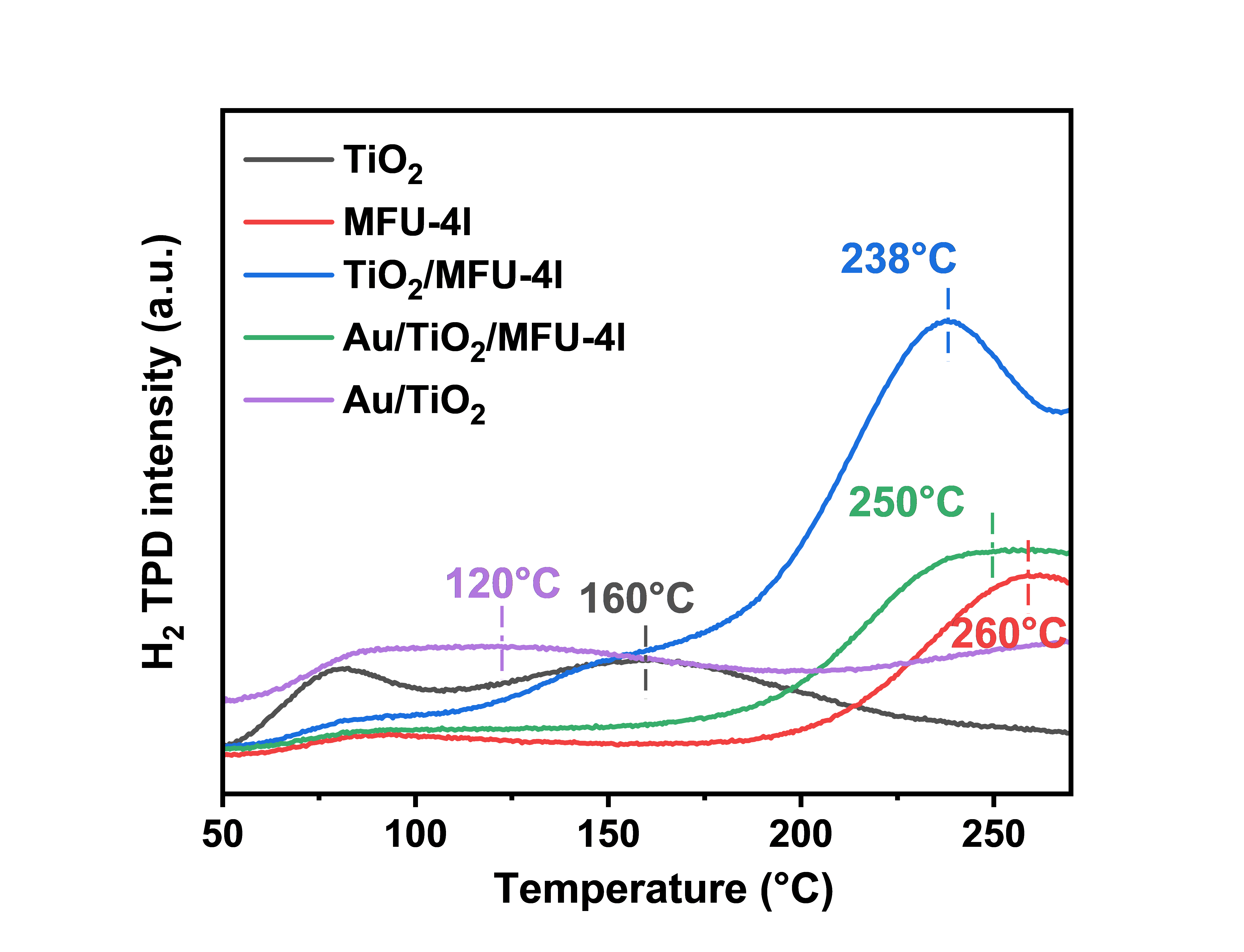


**Figure S24.** H_2_-TPD profiles of different samples.

**Table S1**. Specific surface area and pore size of catalysts.

| Catalyst | Surface area(m^2^/g) | Pore width(nm) |
| --- | --- | --- |
| TiO_2_ | 50.545 | 15.310 |
| MFU-4l | 3429.633 | 1.410 |
| TiO_2_/MFU-4l | 1610.568 | 1.410 |
| Au/TiO_2_/MFU-4l | 480.836 | 3.992 |

**Table S2**. EXAFS fitting parameters at Zn K-edge and Au L3-edge of different samples.

| Zn K-edge | | | | | | |
| --- | --- | --- | --- | --- | --- | --- |
| Sample | Shell | *CN^a^* | *R*(Å)*^b^* | *σ*^2^(Å^2^)*^c^* | Δ*E*_0_(eV)*^d^* | *R* factor |
| Zn foil | Zn-Zn | 6.0* | 2.65±0.01 | 0.0102 | 4.0 | 0.0016 |
|  | Zn-Zn | 6.0* | 2.81±0.01 | 0.0198 | 5.5 |  |
| ZnO | Zn-O | 4.0* | 1.97±0.01 | 0.0036 | 4.9 | 0.0037 |
|  | Zn-Zn | 12.0* | 3.23±0.01 | 0.0080 | 3.4 |  |
|  | Zn-O | 12.0* | 3.71±0.01 | 0.0033 | 0.7 |  |
| ZnCI_2_ | Zn-CI | 4.0* | 2.23±0.01 | 0.0092 | -2.9 | 0.0164 |
| MFU-4l | Zn-O/N | 4.5±0.2 | 2.01±0.01 | 0.0044 | 1.2 | 0.0048 |
|  | Zn-CI | 0.9±0.1 | 2.24±0.01 | 0.0091 | -14.5 |  |
| TiO_2_/MFU-4l | Zn-O/N | 4.9±0.3 | 2.00±0.01 | 0.0058 | 4.6 | 0.0133 |
| Au/TiO_2_/MFU-4l | Zn-O/N | 4.8±0.2 | 2.00±0.01 | 0.0070 | 3.9 | 0.0061 |
| Au L3-edge | | | | | | |
| Sample | Shell | *CN^a^* | *R*(Å)*^b^* | *σ*^2^(Å^2^)*^c^* | Δ*E*_0_(eV)*^d^* | *R* factor |
| Au foil | Au-Au | 12.0* | 2.86±0.01 | 0.0079 | 4.2 | 0.0015 |
| Au_2_O_3_ | Au-O | 4.8±0.2 | 1.98±0.01 | 0.0166 | 9.7 | 0.0062 |
| Au/TiO_2_/MFU-4l | Au-O | 2.4±0.2 | 2.39±0.01 | 0.0062 | -14.8 | 0.0028 |
|  | Au-Au | 7.9±0.2 | 2.85±0.01 | 0.0090 | 3.1 |  |

^a^CN, coordination number; ^b^R, distance between absorber and backscatter atoms; ^c^σ^2^, Debye-Waller factor to account for both thermal and structural disorders; ^d^ΔE_0_, inner potential correction; R factor indicates the goodness of the fit. S_0_^2^ was fixed to 0.843 and 0.751. A reasonable range of EXAFS fitting parameters: 0.700 < S_0_^2^ < 1.000; CN > 0; σ^2^ > 0 Å2; |ΔE_0_| < 15 eV; R factor < 0.02.

**Table S3**. Au content in Au/TiO_2_/MFU-4l measured by ICP-OES.

| Catalyst | Au addition amount/wt % | | Au measurement content/wt % |
| --- | --- | --- | --- |
| 1.6% Au/TiO_2_/MFU-4l | 1.6 | 0.73 | |
| 4% Au/TiO_2_/MFU-4l | 4.0 | 1.30 | |
| 6.4% Au/TiO_2_/MFU-4l | 6.4 | 2.56 | |
| 8% Au/TiO_2_/MFU-4l | 8.0 | 6.17 | |
| 12% Au/TiO_2_/MFU-4l | 12.0 | 6.95 | |

**Table S4**. Performance comparison of recently reported photocatalytic CO_2_ reduction to C_2_H_4_.

| **Catalyst** | **Light range** | | | **Solvent** | **C_2_H_4_ Yield (μmol/g/h)** | | **Selectivity %** | | **Reference** | |
| --- | --- | --- | --- | --- | --- | --- | --- | --- | --- | --- |
| AgInP_2_S_6_ | λ > 420 nm | | | H_2_O | 44.3 | | 73% | | Yujie Xiong, Nat. Commun.2021^[13]^ | |
| Cu_1_/W_18_O_49_ | | λ > 420 nm | | H_2_O | | 4.9 | | 72.8% | | Hefeng Cheng, Adv. Sci. 2024^[14]^ |
| Au Pd/(101) TiO_2_ | | λ > 320 nm | | H_2_O | | 0.7 | | 7% | | Qin Kuang, J. Mater. Chem. A.2019^[15]^ |
| Cu0.8Au0.2/TiO_2_ | | λ > 320 nm | | H_2_O | | 369.8 | | 6.1% | | Fan Dong, ACS Nano. 2021^[10]^ |
| Cu^δ+^ /CeO_2_-TiO_2_ | | λ > 320 nm | | H_2_O | | 4.51 | | 73.9% | | Benxia Li, ACS Nano. 2022^[8]^ |
| MIL-88B-NS40 | | λ > 420 nm | | H_2_O | | 17.7 | | 10.6% | | Fan Guo, Angew. Chem. Int. Ed. 2023^[9]^ |
| CuGaS_2_@CuO | | λ > 400 nm | | H_2_O | | 20.6 | | 75.1% | | Sebastian C. Peter, Angew. Chem. Int. Ed. 2022^[16]^ |
| CuO_X_@p-ZnO | | λ > 420 nm | | H_2_O、TEA | | 2.7、22.3 | | 32.9% | | Hua Sheng, J. Am. Chem. Soc. 2021^[17]^ |
| CuGaS_2_/Ga_2_S_3_ | | λ > 420 nm | | H_2_O、TEOA | | 335.67 | | 93.87% | | Tao Yu, Adv. Funct. Mater. 2023^[18]^ |
| Cu ACs/PCN | | λ > 320 nm | | H_2_O、TEOA、 Ru(bpy)_3_Cl_2_·6H_2_O | | 10.17 | | 53.2% | | Hongwei Huang, Adv. Mater. 2022^[19]^ |
| Cu0.012@CMP | | λ > 420 nm | | MeCN、TEOA | | 236.81 | | 78.2 % | | Jin Wang, Fuel.2024^[20]^ |
| NiAl-Fe-TCPP | | λ > 400 nm | | MeCN、TEOA、H_2_O、Ru(bpy)_3_Cl_2_·6H_2_O | | 2470 | | 93.4% | | Yufei Zhao, Adv. Funct. Mater. 2023^[21]^ |
| Re-bpy/PTF(Cu) | | λ > 400 nm | | MeCN、TEOA | | 73.2 | | 25% | | RongCao, J. Am. Chem. Soc. 2023^[22]^ |
| **Au/TiO_2_/MFU-4l** | | | **λ > 320 nm** | **H_2_O、TEA** | | **107.03** | | **91.54%** | | **This work** |

**References**

[1] M. R. Mian, H. Chen, R. Cao, K. O. Kirlikovali, R. Q. Snurr, T. Islamoglu, O. K. Farha, “Insights into Catalytic Hydrolysis of Organophosphonates at M-OH Sites of Azolate-Based Metal Organic Frameworks” *J. Am. Chem. Soc.* **2021**, *143*, 9893-9900.

[2] C. Chen, M. Wu, Y. Xu, C. Ma, M. Song, G. Jiang, “Efficient Photoreduction of CO_2_ to CO with 100% Selectivity by Slowing Down Electron Transport” *J. Am. Chem. Soc.* **2024**, *146*, 9163-9171.

[3] A. M. Wright, Z. Wu, G. Zhang, J. L. Mancuso, R. J. Comito, R. W. Day, C. H. Hendon, J. T. Miller, M. Dincă, “A Structural Mimic of Carbonic Anhydrase in a Metal-Organic Framework” *Chem.* **2018**, *4*, 2894-2901.

[4] A. W. Stubbs, M. Dincǎ, “Selective Oxidation of C-H Bonds through a Manganese(III) Hydroperoxo in MnII-Exchanged CFA-1” *Inorg. Chem.* **2019**, *58*, 13221-13228.

[5] H. Ou, G. Li, W. Ren, B. Pan, G. Luo, Z. Hu, D. Wang, Y. Li, “Atomically Dispersed Au-Assisted C-C Coupling on Red Phosphorus for CO_2_ Photoreduction to C_2_H_6_.” *J. Am. Chem. Soc.* **2022**, *144*, 22075-22082.

[6] J. Li, D. Ma, Q. Huang, Y. Du, Q. He, H. Ji, W. Ma, J. Zhao, “Cu^2+^ coordination-induced in situ photo-to-heat on catalytic sites to hydrolyze β-lactam antibiotics pollutants in waters” *Proc. Natl. Acad. Sci. U.S.A.* **2023**, *120*, e2302761120.

[7] J. Mao, J. Iocozzia, J. Huang, K. Meng, Y. Lai, Z. Lin, “Graphene aerogels for efficient energy storage and conversion” *Energy Environ. Sci.* **2018**, *11*, 772-799.

[8] T. Wang, L. Chen, C. Chen, M. Huang, Y. Huang, S. Liu, B. Li, “Engineering Catalytic Interfaces in Cu^δ+^ /CeO_2_-TiO_2_ Photocatalysts for Synergistically Boosting CO_2_ Reduction to Ethylene” *ACS Nano* **2022**, *16*, 2306-2318.

[9] F. Guo, R. Li, S. Yang, X. Zhang, H. Yu, J. J. Urban, W. Sun, “Designing Heteroatom-Codoped Iron Metal-Organic Framework for Promotional Photoreduction of Carbon Dioxide to Ethylene” *Angew. Chem. Int. Ed.* **2023**, *62*, e202216232.

[10] Y. Yu, X. Dong, P. Chen, Q. Geng, H. Wang, J. Li, Y. Zhou, F. Dong, “Synergistic Effect of Cu Single Atoms and Au-Cu Alloy Nanoparticles on TiO_2_ for Efficient CO_2_ Photoreduction” *ACS Nano* **2021**, *15*, 14453-14464.

[11] Y. Wu, Q. Chen, J. Zhu, K. Zheng, M. Wu, M. Fan, W. Yan, J. Hu, J. Zhu, Y. Pan, X. Jiao, Y. Sun, Y. Xie, “Selective CO_2_-to-C_2_H_4_ Photoconversion Enabled by Oxygen‐Mediated Triatomic Sites in Partially Oxidized Bimetallic Sulfide” *Angew. Chem. Int. Ed.* **2023**, *62*, e202301075.

[12] S. Gong, Y. Niu, X. Liu, C. Xu, C. Chen, T. J. Meyer, Z. Chen, “Selective CO_2_ Photoreduction to Acetate at Asymmetric Ternary Bridging Sites” *ACS Nano* **2023**, *17*, 4922-4932.

[13] W. Gao, S. Li, H. He, X. Li, Z. Cheng, Y. Yang, J. Wang, Q. Shen, X. Wang, Y. Xiong, Y. Zhou, Z. Zou, “Vacancy-defect modulated pathway of photoreduction of CO_2_ on single atomically thin AgInP_2_S_6_ sheets into olefiant gas” *Nat. Commun.* **2021**, *12*, 4747.

[14] Y. Mao, M. Zhang, G. Zhai, S. Si, D. Liu, K. Song, Y. Liu, Z. Wang, Z. Zheng, P. Wang, Y. Dai, H. Cheng, B. Huang, “Asymmetric Cu(I)-W Dual-Atomic Sites Enable C-C Coupling for Selective Photocatalytic CO_2_ Reduction to C_2_H_4_” *Adv. Sci.* **2024,***11*, 2401933.

[15] Q. Chen, X. Chen, M. Fang, J. Chen, Y. Li, Z. Xie, Q. Kuang, L. Zheng, “Photo-induced Au–Pd alloying at TiO_2_ {101} facets enables robust CO_2_ photocatalytic reduction into hydrocarbon fuels” *J. Mater. Chem. A* **2019**, *7*, 1334-1340.

[16] S. Chakraborty, R. Das, M. Riyaz, K. Das, A. K. Singh, D. Bagchi, C. P. Vinod, S. C. Peter, “Wurtzite CuGaS_2_ with an In-Situ-Formed CuO Layer Photocatalyzes CO_2_ Conversion to Ethylene with High Selectivity” *Angew. Chem. Int. Ed.* **2023**, *62*, e202216613.

[17] W. Wang, C. Deng, S. Xie, Y. Li, W. Zhang, H. Sheng, C. Chen, J. Zhao, “Photocatalytic C-C Coupling from Carbon Dioxide Reduction on Copper Oxide with Mixed-Valence Copper(I)/Copper(II)” *J. Am. Chem. Soc.* **2021**, *143*, 2984-2993.

[18] J. Wang, C. Yang, L. Mao, X. Cai, Z. Geng, H. Zhang, J. Zhang, X. Tan, J. Ye, T. Yu, “Regulating the Metallic Cu-Ga Bond by S Vacancy for Improved Photocatalytic CO_2_ Reduction to C_2_H_4_” *Adv Funct Materials* **2023**, *33*, 2213901.

[19] W. Xie, K. Li, X. Liu, X. Zhang, H. Huang, “P‐Mediated Cu–N_4_ Sites in Carbon Nitride Realizing CO2 Photoreduction to C2 H4 with Selectivity Modulation” *Adv. Mater.* **2023**, *35*, 2208132.

[20] J. Li, T. Chan, C. Jing, J. Wang, “CO_2_ photoreduction with high selectivity of C_2_H_4_ production on conjugated microporous polymer with Cu single atoms” *Fuel* **2024**, *357*, 130020.

[21] C. Ning, J. Yang, S. Bai, G. Chen, G. Liu, T. Shen, L. Zheng, S. Xu, X. Kong, B. Liu, Y. Zhao, Y. Song, “An Efficient Intercalation Supramolecular Structure for Photocatalytic CO2 Reduction to Ethylene Under Visible Light” *Adv. Funct. Mate.* **2023**, *33*, 2300365.

[22] R. Xu, D.-H. Si, S.-S. Zhao, Q.-J. Wu, X.-S. Wang, T.-F. Liu, H. Zhao, R. Cao, Y.-B. Huang, “Tandem Photocatalysis of CO_2_ to C_2_H_4_ via a Synergistic Rhenium-(I) Bipyridine/Copper-Porphyrinic Triazine Framework” *J. Am. Chem. Soc.* **2023**, *145*, 8261–8270.
